# Methionine deficiency inhibited pyroptosis in primary hepatocytes of grass carp (*Ctenopharyngodon idella*): possibly via activating the ROS-AMPK-autophagy axis

**DOI:** 10.1186/s40104-024-01069-6

**Published:** 2024-09-02

**Authors:** Yuanlin He, Pei Wu, Weidan Jiang, Yang Liu, Xiaowan Jin, Hongmei Ren, Ruinan Zhang, Xiaoqiu Zhou, Lin Feng

**Affiliations:** 1 Animal Nutrition Institute, Sichuan Agricultural University, Chengdu, 611130 Sichuan China; 2 Fish Nutrition and Safety Production, University Key Laboratory of Sichuan Province, Sichuan Agricultural University, Chengdu, 611130 Sichuan China; 3 Key Laboratory of Animal Disease-Resistant Nutrition and Feed, Ministry of Agriculture and Rural Affairs, Chengdu, China; 4 Key Laboratory of Animal Disease-Resistant Nutrition, Chengdu, Sichuan China

**Keywords:** Autophagy, Methionine, Primary hepatocytes, Pyroptosis

## Abstract

**Background:**

Methionine (Met) is the only sulfur-containing amino acid among animal essential amino acids, and methionine deficiency (MD) causes tissue damage and cell death in animals. The common modes of cell death include apoptosis, autophagy, pyroptosis, necroptosis. However, the studies about the major modes of cell death caused by MD have not been reported, which worth further study.

**Methods:**

Primary hepatocytes from grass carp were isolated and treated with different doses of Met (0, 0.5, 1, 1.5, 2, 2.5 mmol/L) to examine the expression of apoptosis, pyroptosis, autophagy and necroptosis-related proteins. Based on this, we subsequently modeled pyroptosis using lipopolysaccharides and nigericin sodium salt, then autophagy inhibitors chloroquine (CQ), AMP-activated protein kinase (AMPK) inhibitors compound C (CC) and reactive oxygen species (ROS) scavengers *N*-acetyl-L-cysteine (NAC) were further used to examine the expression of proteins related to pyroptosis, autophagy and AMPK pathway in MD-treated cells respectively.

**Results:**

MD up-regulated B-cell lymphoma protein 2 (Bax), microtubule-associated protein 1 light chain 3 II (LC3 II), and down-regulated the protein expression levels of B-cell lymphoma-2 (Bcl-2), sequestosome 1 (p62), cleaved-caspase-1, cleaved-interleukin (IL)-1β, and receptor-interacting protein kinase (RIP) 1 in hepatocytes, while it did not significantly affect RIP3. In addition, MD significantly increased the protein expression of liver kinase B1 (LKB1), p-AMPK, and Unc-51-like kinase 1 (ULK1) without significant effect on p-target of rapamycin. Subsequently, the use of CQ increased the protein expression of NOD-like receptor thermal protein domain associated protein 3 (NLRP3), cleaved-caspase-1, and cleaved-IL-1β inhibited by MD; the use of CC significantly decreased the protein expression of MD-induced LC3 II and increased the protein expression of MD-suppressed p62; then the use of NAC decreased the MD-induced p-AMPK protein expression.

**Conclusion:**

MD promoted autophagy and apoptosis, but inhibited pyroptosis and necroptosis. MD inhibited pyroptosis may be related regarding the promotion of autophagy. MD activated AMPK by inducing ROS production which in turn promoted autophagy. These results could provide partial theoretical basis for the possible mechanisms of Met in ensuring the normal structure and function of animal organs. Furthermore, ferroptosis is closely related to redox states, it is worth investigating whether MD affects ferroptosis in hepatocytes.

**Supplementary Information:**

The online version contains supplementary material available at 10.1186/s40104-024-01069-6.

## Introduction

Methionine (Met) is not only one of the essential amino acids for animals, but also the only sulfur-containing amino acid, which plays an extremely critical part in the growth and development of animals [1, 2]. After Met absorption into the animals, it is metabolized mainly in the liver, which is the largest organ of detoxification and nutrient metabolism in animals [3]. Some studies have found that dietary methionine deficiency (MD) resulted in swelling, blunted edges and yellow coloration of broiler livers [4]. Moreover, MD increased chromatin margination and nuclear curling in the liver of *Pelteobagrus fulvidraco*, which no longer had the typical appearance of the nucleus and nucleolus of hepatocytes [5]. In fact, excessive cell death also contributes to tissue and organ damage [6]. Currently, the common modes of cell death include apoptosis, autophagy, pyroptosis, necroptosis and so on [7]. However, studies about the major modes of cell death caused by MD have not been reported.

To date, most of the research on MD and cell death focuses on apoptosis. Studies have found that MD boosted the gene expression of B-cell lymphoma protein 2 (*Bax*) and *caspase-3* and reduced the expression of B-cell lymphoma-2 (*Bcl-2*) in the kidney of Cobb broiler chicks [8]; and enhanced the number of apoptotic cells in HepG2 cells as well as in hepatocytes of *P. fulvidraco* [5]. Meanwhile, autophagy is a procedure in which eukaryotic cells utilize lysosomes to degrade cellular damaged organelles and proteins [9]. Autophagy begins with the formation of phagosomes; then the lipid-soluble microtubule-associated protein light chain 3 (LC3) II binds to the phagosome to constitute an autophagosome; ultimately, the autolysosome, which is formed by the combination of autophagosome and lysosome, degrades the material needed to be degraded or cleared [10]. However, studies on MD and autophagy have not yet been reported in fish. Only one study has been shown that dietary Met-choline deficiency increased protein expression levels of LC3 II in the liver of male mice [11]. In addition, it was found that MD increased the gene expression of AMP-activated protein kinase (*AMPK*) in the hepatocytes of *P. fulvidraco*, and supplementation of Met increased the protein expression of target of rapamycin (TOR) in bovine mammary epithelial cells [5, 12]. In turn, the AMPK-TOR pathway can be involved in regulating autophagy in yeast [13]. Therefore, MD may affect autophagy, which need to be further investigated.

Moreover, some studies have shown that MD also increased lipopolysaccharide (LPS)-induced inflammation in RAW 264.7 macrophages [14]. Among all modes of death, pyroptosis and necroptosis are accompanied by inflammation [15]. Yet studies on MD and pyroptosis have not been reported. Pyroptosis is dependent on inflammatory caspases (including caspase-1/4/5/11) and the gasdermin (GSDM) protein family (including GSDMA-E and DFNB 59) [16]. During pyroptosis, activation of inflammasomes further activates caspase-1, which can cleave GSDM proteins and the precursors of interleukin (IL)-1β, the former forms pore in cell membranes and the latter is released from the pores then amplifies the inflammatory response [17, 18]. Currently, existing studies on pyroptosis have focused on the NOD-like receptor thermal protein domain associated protein 3 (NLRP3)-dependent pathway; and only one gasdermin E (GSDME), has been identified in teleost fish [19–21]. We knew that one of the functions of Met is to synthesize glutathione (GSH), which scavenges reactive oxygen species (ROS) and mitigates oxidative damage. In recent years, it has been noted that dietary MD increased the content of ROS in male rat liver [22] and reduced the content of GSH in mouse liver [23]. In addition, it has been found that the ROS markedly enhanced the expression of NLRP3 in human umbilical vein endothelial cells as well as in RSC96 cells [24, 25]. As a result, the relationship between MD and pyroptosis have not been reported, which remains to be investigated.

Also, grass carp (*Ctenopharyngodon idella*) is the most abundant freshwater fish in China and dominates global inland aquaculture [26]. Consequently, we explored the relationship between MD and cell death which focused on autophagy and pyroptosis using primary hepatocytes of grass carp as a research model. To supply theoretical evidence for enriching the nutritional physiological functions of Met.

## Materials and methods

### Antibodies and reagents information

The following antibodies were used in the experiments: anti-NLRP3 (A5652, 1:1,000, ABclonal, Hubei, China), anti-cleaved-caspase-1 (AF4005, 1:1,000, Affinity, Jiangsu, China), anti-GSDME (A7432, 1:1,000, ABclonal, Hubei, China), anti-N-GSDME (AF4016, 1:1,000, Affinity), anti-cleaved-IL-1β (AF4006, 1:1,000, Affinity), anti-Bax (AF0120, 1:1,000, Affinity), anti-Bcl-2 (AF6139, 1:1,000, Affinity), anti-sequestosome 1 (p62) (A7758, 1:1,000, ABclonal), anti-LC3 II (A5681, 1:1,000, ABclonal), anti-receptor-interacting protein kinase (RIP) 1 (A7414, 1:1,000, ABclonal), anti-RIP3 (A5431, 1:1,000, ABclonal), anti-liver kinase B1 (LKB1) (A2122, 1:1,000, ABclonal, Hubei, China), anti-p-AMPK (AP0871, 1:1,000, ABclonal, Hubei, China), anti-p-TOR (AP0928, 1:1,000, ABclonal, Hubei, China), anti-Unc-51-like kinase 1 (ULK1) (A8529, 1:1,000, ABclonal, Hubei, China). The following reagents were used in the experiments: LPS (L2880, Sigma-Aldrich, St. Louis, Missouri, USA), NLRP3 activator nigericin sodium salt (Nig) (S6653, Selleck Chemicals, Houston, TX, USA), autophagy inhibitor chloroquine (CQ) (S6999, Selleck Chemicals), AMPK inhibitor compound C (CC) (S7840, Selleck Chemicals), ROS scavenger *N*-acetyl-L-cysteine (NAC) (T0875, TargetMol Chemicals, Boston, MA, USA).

### Histological examination

A total of 540 grass carp (178.47 ± 0.36 g) were selected and fed six diets with different Met concentrations (2.54, 4.85, 7.43, 10.12, 12.40 and 15.11 g/kg diet, experimental diets was showed in Table S1) for 60 d, and the liver were collected for the subsequent experiments, which is identical to Fang et al. [27]. Liver samples packed in 4% paraformaldehyde solution had been dehydrated and inserted in a paraffin wax. The tissue block was then cut into 4 μm thick tissue slices using a paraffin cutter. Tissue sections were observed and photographed under a Nikon TS100 light microscope after being stained with hematoxylin and eosin (H&E).

### Cell culture and identification

After sterilized the fish, the livers were removed, cleaned and minced, then added with 0.25% trypsin and digested for 20 min at 28 °C in a constant temperature shaker at low speed. After digestion was completed, medium containing 10% fetal bovine serum (FBS) was added to terminate the digestion. The cell filtrate is then collected and centrifuged at 1,000 × *g* for 1 min; the supernatant is discarded and phosphate buffer saline (PBS) and erythrocyte lysate are added in a 1:3 ratio; centrifuged at 1,000 × *g* for 3 min; the supernatant is discarded and centrifuged at 1,000 × *g* for 1 min, followed by a final centrifugation at 500 × *g* for 1 min. Our isolation method was referenced from Shi et al. [28]. Finally, complete medium (leibovitz’s L-15 (L15) + 10% FBS + 1% antibiotic-antimycobacterial solution + 10 μg/mL insulin + 40 μg/mL transferrin + 2 mmol/L glutamine) was added to the cells then the cells were inoculated into cell culture plates at a rate of 1–2 × 10^6^ cells/mL and incubated in 28 °C for 48 h), and cells were subsequently characterized by morphology and hepatocyte-specific protein expression of albumin (ALB) [29].

### Experimental design

In order to identify the primary hepatocytes from grass carp, replace with new L15 medium and treated cells for 24, 48, 72, 96, 120, 144 h. To screen for the optimal dose and treatment time of Met, change the treatment solution to (L15 + 10% FBS + 1% antibiotic-antimycobacterial solution with various concentrations of Met (0, 0.5, 1, 1.5, 2, 2.5 mmol/L) and continue to culture the cells for 24, 48 or 72 h. Subsequently, 0 or 1 mmol/L Met was selected to treat cells for 48 h to investigate the effect of MD on cell death. To establish a model of pyroptosis, cells were treated with L15 medium containing 100 ng/mL LPS for 4 h directly, followed replaced with L15 containing the various concentrations of Nig (0, 0.5, 1, 2, 4, 8 μmol/L) for 0.5 h. Afterwards, cells were treated with 0 or 1 mmol/L Met for 48 h, then treated with LPS, and finally treated with 0.5 μmol/L Nig for 0.5 h, to investigate the effect of MD on pyroptosis. Toward exploring the effect of MD on pyroptosis via autophagy, we co-treated cells for 48 h by adding different concentrations of CQ (5, 10, 20, 40 μmol/L) to the medium. For the purpose of exploring the effect of MD on autophagy via AMPK, we co-treated the cells for 48 h by adding different concentrations of CC (2, 4, 8, 16 μmol/L) to the medium. As to explore the effect of MD on AMPK via ROS, we co-treated cells for 48 h by adding different concentrations of NAC (0, 2.5, 5, 7.5 mmol/L) to the medium.

### Cell viability assay

Then cell viability was tested by using the Cell Counting Kit-8 (CCK-8) (C0005, Taoshu, Shanghai, China) as described by the manufacturer. In brief, hepatocytes were cultured to reach the desired confluence in 96-well plates. Then, hepatocytes were incubated with different Met treatment for 48 h, then adding 10 μL CCK-8 to each well. Following 2–3 h incubation, a microplate reader (Tecan, Mannedorf, Switzerland) with an excitation wavelength of 450 nm was used to detect the absorbance.

### ALB content assay

The culture supernatant was collected after the cell treatment and the ALB content detection kit (A028, Nanjing Jiancheng, Jiangsu, China) was used to detect the ALB content as described by the manufacturer. The absorbance of the sample was measured by a microplate reader (Tecan, Mannedorf, Switzerland) with an excitation wavelength of 630 nm.

### Lactate dehydrogenase (LDH) release assay

The culture supernatants were harvested and the levels of LDH were determined using the LDH cytotoxicity detection kit (A020, Nanjing Jiancheng, Jiangsu, China) as described by the manufacturer. The microplate reader (Tecan, Mannedorf, Switzerland) with an excitation wavelength of 450 nm was used to measure the absorbance of samples.

### Hoechst/propidium iodide (PI) staining

Hoechst/PI staining was carried out on hepatocytes by utilizing the Hoechst 33342/PI staining kit (CA1120, Solarbio, Beijing, China). Cells were removed and rinsed with PBS, then stained with Hoechst 33342 and PI staining solution for 30 min at 4 °C. Finally, the cells were washing with PBS and observed with a biomicroscope (DMI 4000 B, Leica, Germany).

### GSH content assay

Based on the manufacturer’s instructions (A006, Nanjing Jiancheng, Jiangsu, China), the cells were cleaned with PBS twice, the cell precipitation was collected by centrifugation at low speed, the cells were broken by ultrasound, and the supernatant was then centrifuged for subsequent reaction. The absorbance of the sample was measured by a microplate reader with an excitation wavelength of 405 nm.

### ROS production determination

ROS production was measured by ROS detection kit (S0033S, Beyotime Biotechnology, Shanghai, China). Cells were first cleaned with PBS, followed by the addition of culture medium containing 10 μmol/L 2′,7′-dichlorodihydrofluorescein diacetate (DCFH-DA), and placed at 37 °C for 30 min, protected from light. The cells were then cleaned with serum-free medium and the staining result was imaged using a biomicroscope.

### Transmission electron microscope (TEM)

After cells were collected from each treatment group, 4% paraformaldehyde were added to cells in room temperature and fixed without light for 30 min, and then transferred to Sevierbo (Hubei, China) for cell morphology photography.

### Immunofluorescence staining

Cells were washed and fixed with 4% tissue cell fixative for 15 min, permeabilized with Triton X-100 for 30 min, and then blocked with goat serum for 30 min. Then, the cells were incubated overnight at 4 °C with primary antibody dilution. The next day, cells were washed with PBS and incubated with FITC-conjugated secondary antibody for 60 min in the dark. Finally, the nuclei were stained with DAPI (C0065, Solarbio, Beijing, China) for 5 min and quenchers were added before imaging using a laser scanning confocal microscope (LSM 800, Zeiss, Germany).

### RT-qPCR analysis

The expression of relevant genes was detected by real-time fluorescence quantitative PCR. Total RNA was obtained using the Total RNA Extraction Kit (RE-03111, Foregene, Sichuan, China). The quantity and quality of extracted RNA were subsequently detected by 1% agarose gel electrophoresis and spectrophotometry (260:280 nm ratio) [30]. Subsequently, RNA was reverse transcribed and cDNA was synthesized using the cDNA Reverse Transcription Kit (RT-01022, Foregene, Sichuan, China). cDNA was then added with specific amplification primers, fluorescent dyes, and double-distilled water, and was mixed and denatured on a CFX96TM Real-Time System fluorescence quantitative PCR instrument. The housekeeping gene was β-actin [31]. The annealing temperatures and specific primer sequences used are given in Table 1. The relative expression of mRNA was calculated with reference to Zhao et al. [32] using the 2^−ΔΔCT^ method.

**Table 1:** Real-time PCR primer sequences

Target gene	Primer sequence forward (5′→3′)	Primer sequence reverse (5′→3′)	Accession number
*NLRP3*	CAGCGGCGGCCAATC	TCCGCGTACCTCCGTGAA	MW767970
*ASC*	TGGCGCGGGTCCTGTA	TCCGCTGCCAGTTCATGAC	MW767971
*Caspase-1*	ACGTCTTGCCCTGCTTATCAAC	CGCCCCTCTCCTGGTCATA	KX231773
*GSDME*	GCTTTTGTGCACTGGCTGACT	GAATTTTCCTCAACAGAAGCAGGAT	MT513755
*IL-1β*	AGAGTTTGGTGAAGAAGAGG	TTATTGTGGTTACGCTGGA	JQ692172
β-actin	GGCTGTGCTGTCCCTGTA	GGGCATAACCCTCGTAGAT	M25013

*NLRP3* NOD-like receptor thermal protein domain associated protein 3, *ASC* Apoptosis-associated speck-like protein containing a CARD, *Caspase-1* Cysteinyl aspartate specific proteinase-1, *GSDME* Gsdermin E, *IL-1β *Interleukin-1β

### Western blot (WB)

The proteins were first divided through SDS-PAGE gel electrophoresis, after that, they were transcribed onto PVDF membranes by a wet method. After the transfer was completed, the PVDF membrane was immersed in bovine serum albumin solution (5%, 1.5 h) on room temperature, after that, it was incubated overnight at 4 °C in a refrigerator. It was cleaned with TBST solution after primary antibody incubation, then followed by incubation with goat anti-rabbit secondary antibody about 1 h [33]. Then, the membranes were cleaned and photographed, followed by quantitative analysis by NIH Image 1.63 software using ECL reagent (PD204, Oriscience Biotechnology, Sichuan, China) as a chromogenic agent. Protein bands were quantitated through ImageJ (NIH, USA).

### Calculations and statistical analysis

All data were given as mean ± standard deviation (SD). A one-way ANOVA tests or independent samples *t*-test were carried out using SPSS 25.0 (SPSS Inc., Chicago, USA) combined with Duncan’s method for multivariable comparisons, and differences were assumed to be significant if *P* < 0.05.

## Results

### Influence of dietary MD on the liver pathology of grass carp

After a 60-d growth test, we observed tissue sections of grass carp livers. As a result, the liver in the MD group showed ruptured cell membranes and severe congestion of hepatic sinusoids (Fig. S1A); while the liver in the optimal group were well defined and structurally intact (Fig. S1B).

### Identification of primary hepatocytes of grass carp

**Fig. 1:**
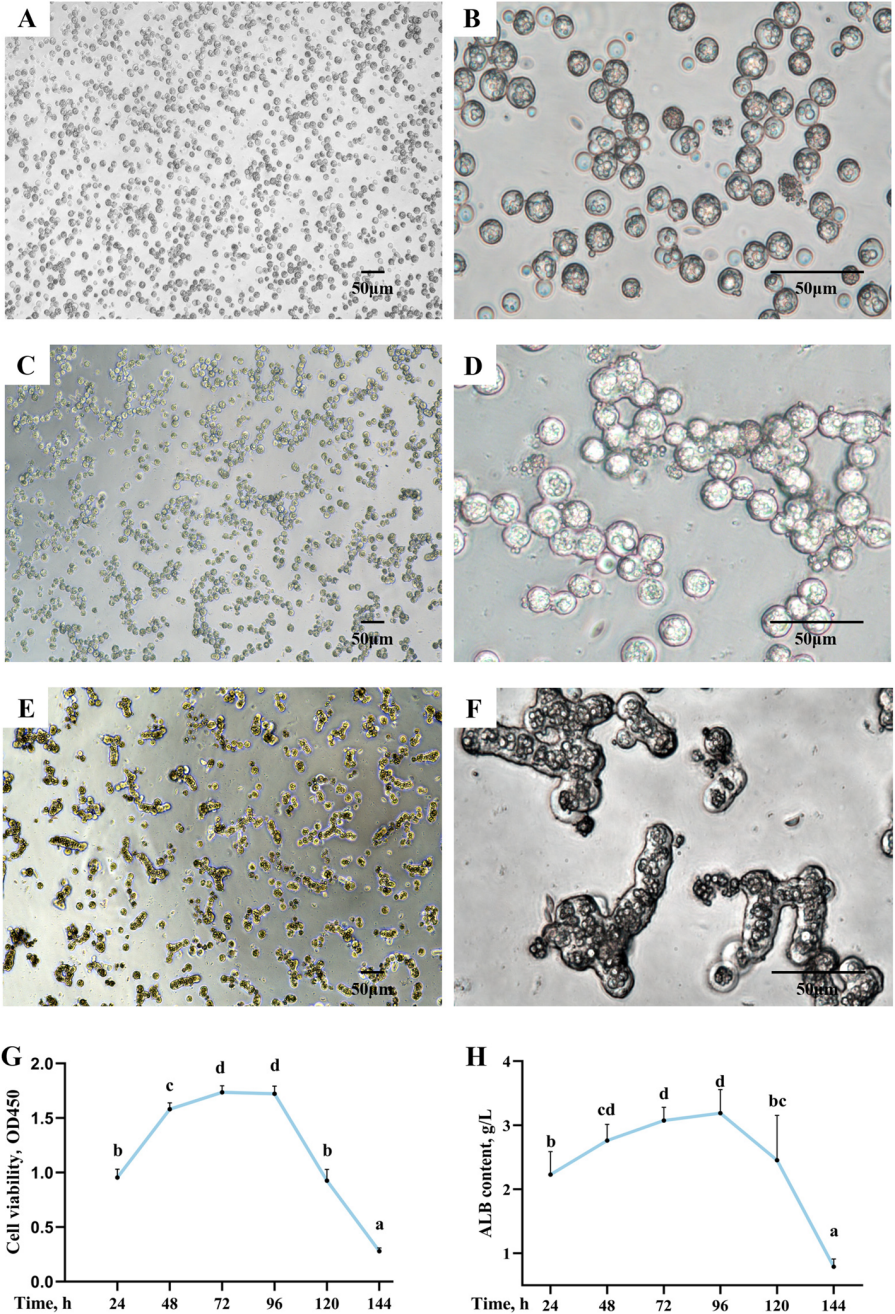
Isolation and culture of primary hepatocytes of grass carp. Freshly digested grass carp primary hepatocytes (100 ×) (**A**) and (400 ×) (**B**). Primary hepatocytes of grass carp grown for 24 h (100 ×) (**C**) and (400 ×) (**D**). Grass carp primary hepatocytes (100 ×) grown for 48 h (**E**) and (400 ×) (**F**). Growth curve (**G**). ALB contents (**H**) of primary hepatocytes of grass carp for growing different times (24, 48, 72, 96, 120, 144 h). The results were expressed as mean ± SD of 6 independent observations. ^a^^–^^d^Values having different letters are significantly different (*P* < 0.05). ALB, albumin

Freshly digested primary hepatocytes of grass carp were circular in shape with intact cell membranes and subcircular nuclei located on the left side of the cells (Fig. 1A and B). Round and subrounded cells are hepatic parenchymal cells that contain hepatic acinus. After 24 h of culture, the cells began to grow in sheets (Fig. 1C and D). At this time, several cells joined together and grew into chains. 48 h later, the cells enlarged, and the fusion of phase cells were obvious (Fig. 1E and F). And this data results suggested that the viability of cells grown for 72–96 h were significantly higher than that of cells grown for 120–144 h, and the same trend was observed for ALB levels (Fig. 1G and H, *P* < 0.05).

### Screening of culture concentration and time by Met treatment in primary hepatocytes of grass carp

**Fig. 2:**
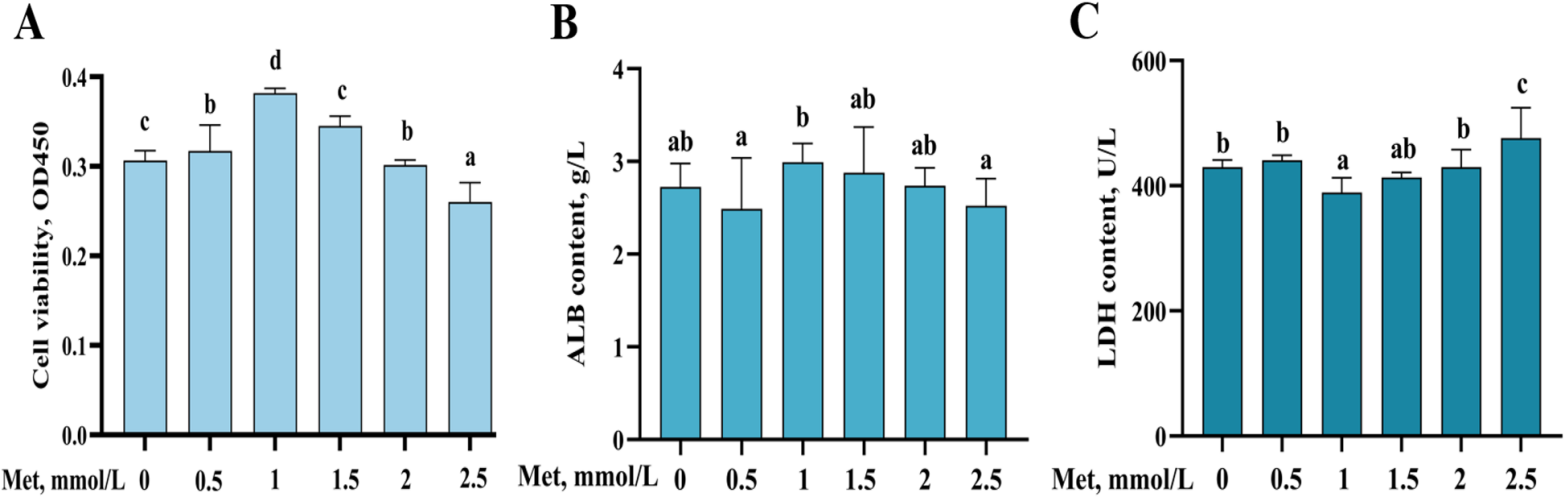
Effect of Met on primary hepatocytes of grass carp. Cell viability (**A**), ALB expression (**B**), and LDH content (**C**) of grass carp primary hepatocytes treated with different concentrations of Met for 48 h. The results were expressed as mean ± SD of 6 independent observations. ^a–d^Values having different letters are significantly different (*P* < 0.05). Met, methionine; ALB, albumin; LDH, lactate dehydrogenase

After treated the cells with different concentrations of Met for 48 h, this result found that the cell viability was significantly higher in the Met concentration of 1 mmol/L than in the MD group (Fig. 2A, *P* < 0.05). The content of ALB had the same trend with cell viability (Fig. 2B, *P* < 0.05). In addition, the activity of LDH in the cell culture medium of the MD group was marked greater than that of the 1 mmol/L Met group (Fig. 2C, *P* < 0.05). In summary, the result determined that the optimal Met supplemental level for hepatocyte growth was 1 mmol/L.

### Effect of MD on primary hepatocyte death of grass carp

**Fig. 3:**
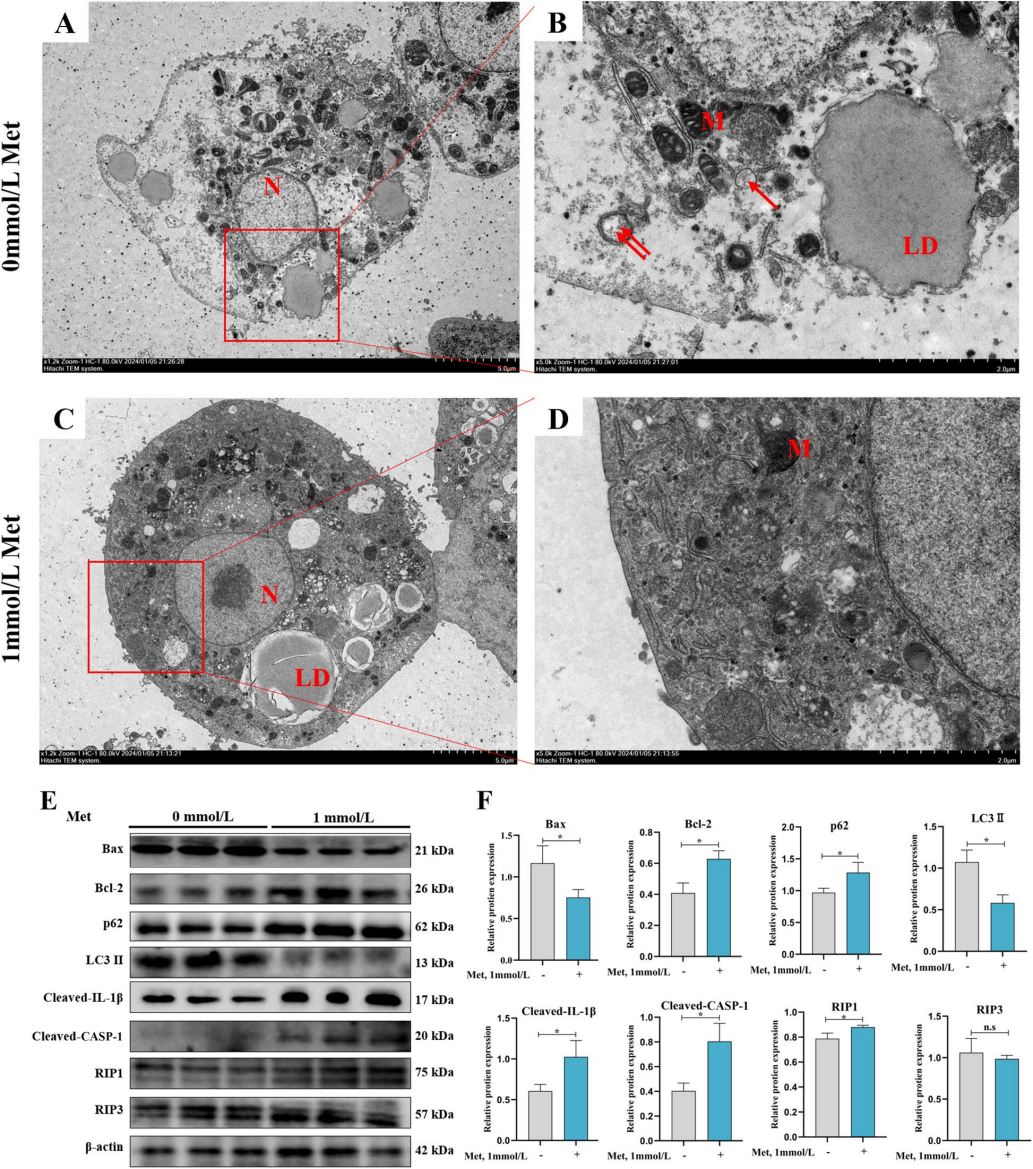
Effect of MD on primary hepatocyte death of grass carp. Observation of primary hepatocytes of grass carp by TEM: MD group (1,200 ×) (**A**) and (5,000 ×) (**B**); control group (1,200 ×) (**C**) and (5,000 ×) (**D**); Relative protein expression of Bax, Bcl-2, p62, LC3 II, cleaved-IL-1β, cleaved-caspase-1, RIP1, RIP3 (**E**) and quantification (**F**) after with or without 1 mmol/L Met treatment for 48 h. The results were expressed as mean ± SD of 3 independent observations. ^*^P < 0.05, n.s: no significance. Met, methionine; N, nucleus; M, mitochondrion; LD, lipid droplet; Bax, B-cell lymphoma protein 2; Bcl-2, B-cell lymphoma-2; p62, sequestosome 1; LC3, microtubule-associated protein 1 light chain 3; IL-1β, interleukin-1β; CASP-1, cysteinyl aspartate specific proteinase-1; RIP1, receptor-interacting protein kinase 1; RIP3, receptor-interacting protein kinase 3

After treating cells with MD and Met supplementation (1 mmol/L) for 48 h, it was found that MD significantly increased chromatin fragmentation in the nucleus of cells and rupture of cell membranes and an autophagosome-like structure appeared in the cell (red arrow) (Fig. 3A–D). Moreover, it marked enhanced the protein expression of Bax, LC3 II and reduced the protein expression of Bcl-2, p62, cleaved-caspase-1, cleaved-IL-1β and RIP1 in cells, while it had no significant effect on RIP3 (Fig. 3E and F, *P* < 0.05).

### Construction of pyroptosis model of primary hepatocytes of grass carp

**Fig. 4:**
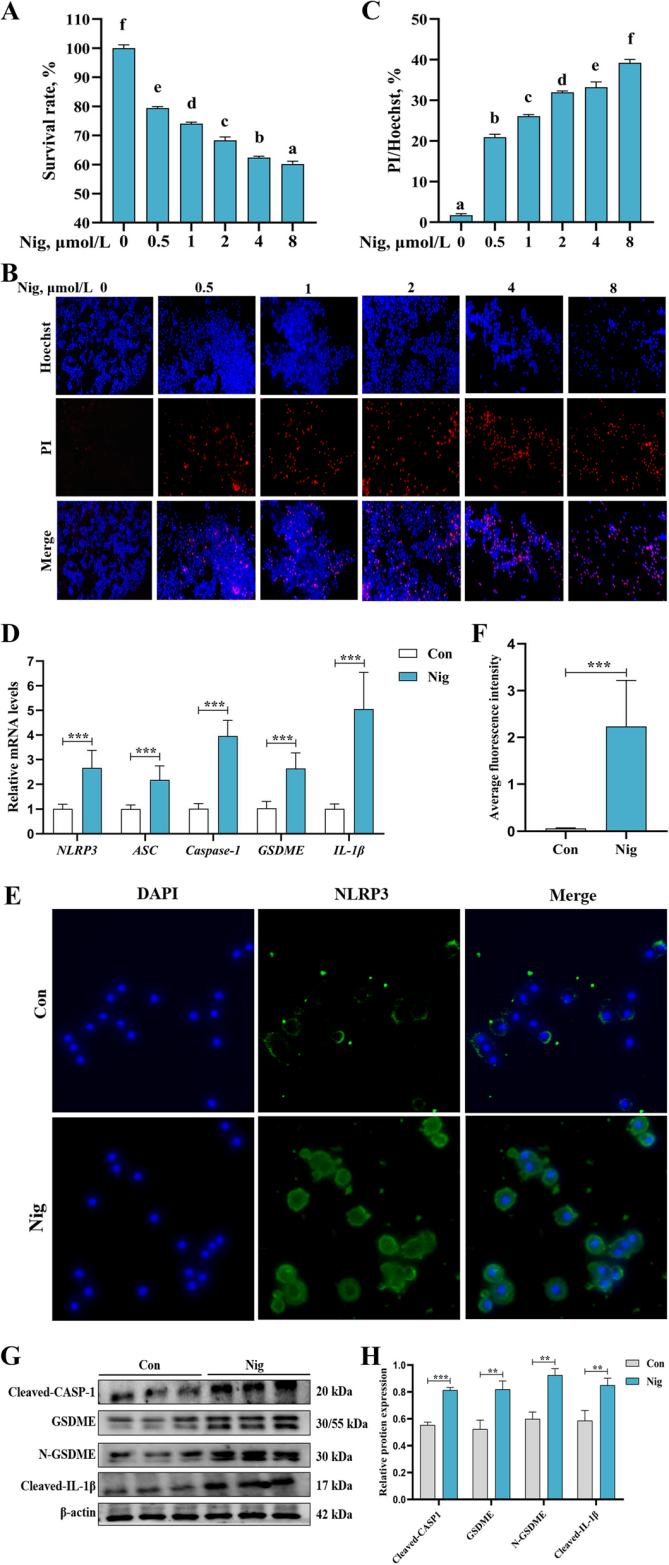
Construction of pyroptosis model using primary hepatocytes of grass carp. Cell viability (**A**), Hoechst/PI staining (**B**), and quantification (**C**) after 100 ng/mL LPS treatment for 4 h, followed by different concentrations of Nig treatment for 0.5 h. Gene expression of *NLRP3*, *ASC*, *caspase-1*, *GSDME*, and *IL-1β* (**D**); NLRP3 immunofluorescence staining (**E**) and quantification (**F**). Protein expression of cleaved-caspase-1, GSDME, N-GSDME, IL-1β (**G**) and quantification (**H**) in these two groups. N: Nucleus. The results were expressed as mean ± SD of 3 or 6 independent observations (WB: *n* = 3). ^a^^–^^f^Values having different letters are significantly different (*P* < 0.05); ^**^P < 0.01, ^***^P < 0.001. Nig, nigericin sodium salt; Con, control; NLRP3, NOD-like receptor thermal protein domain associated protein 3; ASC, apoptosis-associated speck-like protein containing a CARD; CASP-1, cysteinyl aspartate specific proteinase-1; GSDME, gasdermin E; IL-1β, interleukin-1β

After treat with LPS and Nig for the specified time and dose, this result found that with the increase of Nig concentration, the cell viability decreased in a dose-dependent manner, and the survival rate was about 80% at 0.5 μmol/L Nig (Fig. 4A, *P* < 0.05). Similarly, after Hoechst/PI staining, the mortality rate of the cells increased progressively with increasing Nig dose, and with a 20% mortality rate at 0.5 μmol/L Nig (Fig. 4B and C, *P* < 0.05). Therefore, 0.5 μmol/L was selected for the construction of pyroptosis in subsequent tests. The gene expression of *NLRP3*, apoptosis-associated speck-like protein containing a CARD (*ASC*), *caspase-1*, *GSDME* and *IL-1β* in the pyroptosis group were significantly increased (Fig. 4D, *P* < 0.05). Also, the protein expression of NLRP3, cleaved-caspase-1, GSDME, N-GSDME and cleaved-IL-1β in the pyroptosis group were significantly increased (Fig. 4E–H, *P* < 0.05). Thus, the pyroptosis model is established successfully.

### Effect of MD on pyroptosis of primary hepatocytes of grass carp

**Fig. 5:**
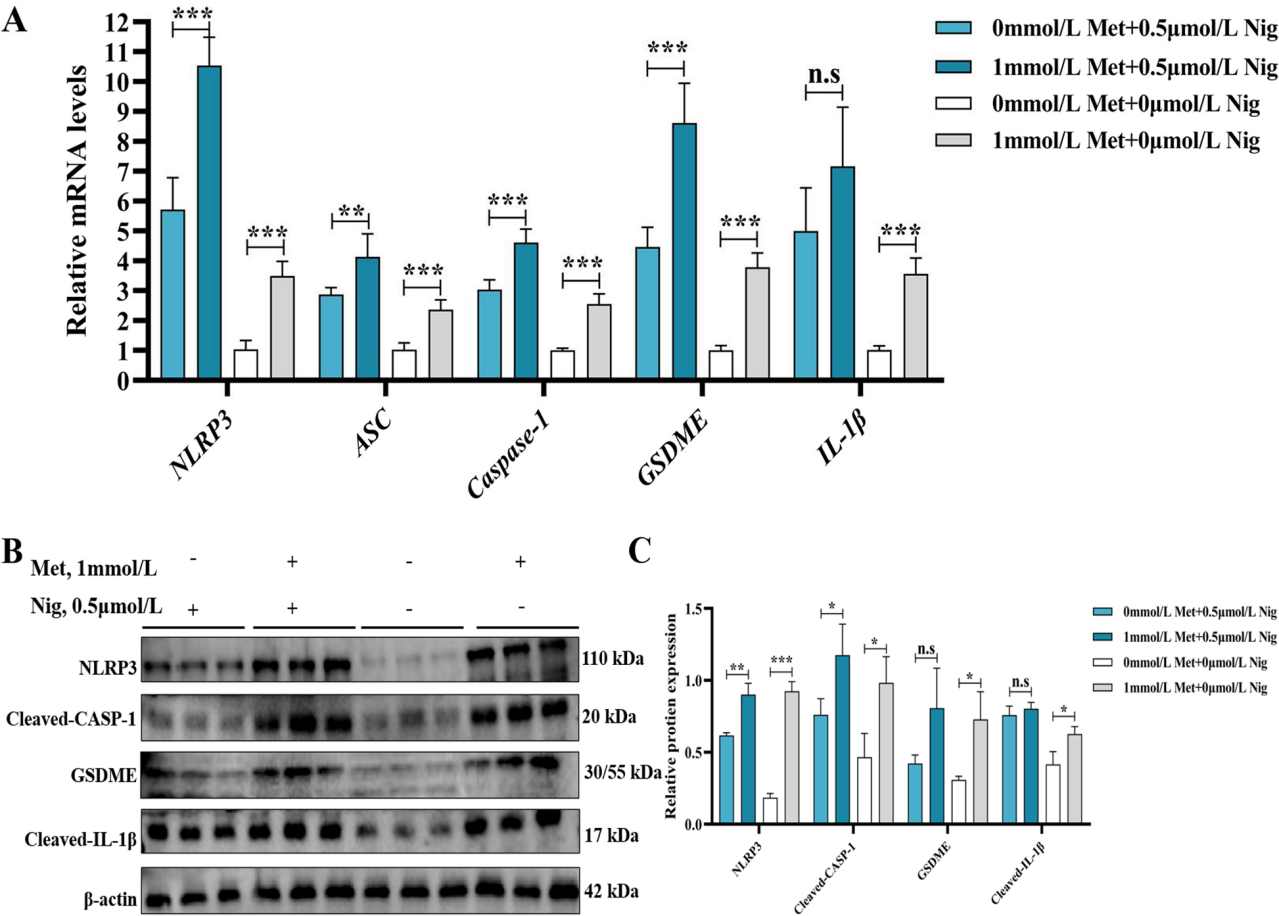
Effect of Met on pyroptosis of primary hepatocytes of grass carp. Gene expression of *NLRP3*, *ASC*, *caspase-1*, *GSDME*, *IL-1β* (**A**); relative protein expression of NLRP3, cleaved-caspase-1, GSDME, cleaved-IL-1β (**B**) and quantification (**C**) after with or without 1 mmol/L Met treatment for 48 h followed by 100 ng/mL lipopolysaccharide (LPS) treatment for 4 h and then 0.5 μmol/L Nig treatment for 0.5 h. The results were expressed as mean ± SD of 3 or 6 independent observations (WB: *n* = 3). ^*^P < 0.05, ^**^P < 0.01, ^***^P < 0.001, n.s: no significance. Met, methionine; Nig, nigericin sodium salt; NLRP3, NOD-like receptor thermal protein domain associated protein 3; ASC, apoptosis-associated speck-like protein containing a CARD; CASP-1, cysteinyl aspartate specific proteinase-1; GSDME, gasdermin E; IL-1β, interleukin-1β

Cells were first treated with Met and then Nig. It turned out that the gene expression of *NLRP3*, *ASC*, *caspase-1*, *GSDME* and *IL-1β* in the MD group were marked lower than that in the 1 mmol/L Met group (Fig. 5A, *P* < 0.05). Protein expression of NLRP3, cleaved-caspase-1. GSDME and cleaved-IL-1β were also found to be significantly lower (Fig. 5B and C, *P* < 0.05). From this, the result concluded that the probability of pyroptosis was lower in the MD group.

### MD inhibits pyroptosis of primary hepatocytes of grass carp through increasing autophagy

**Fig. 6:**
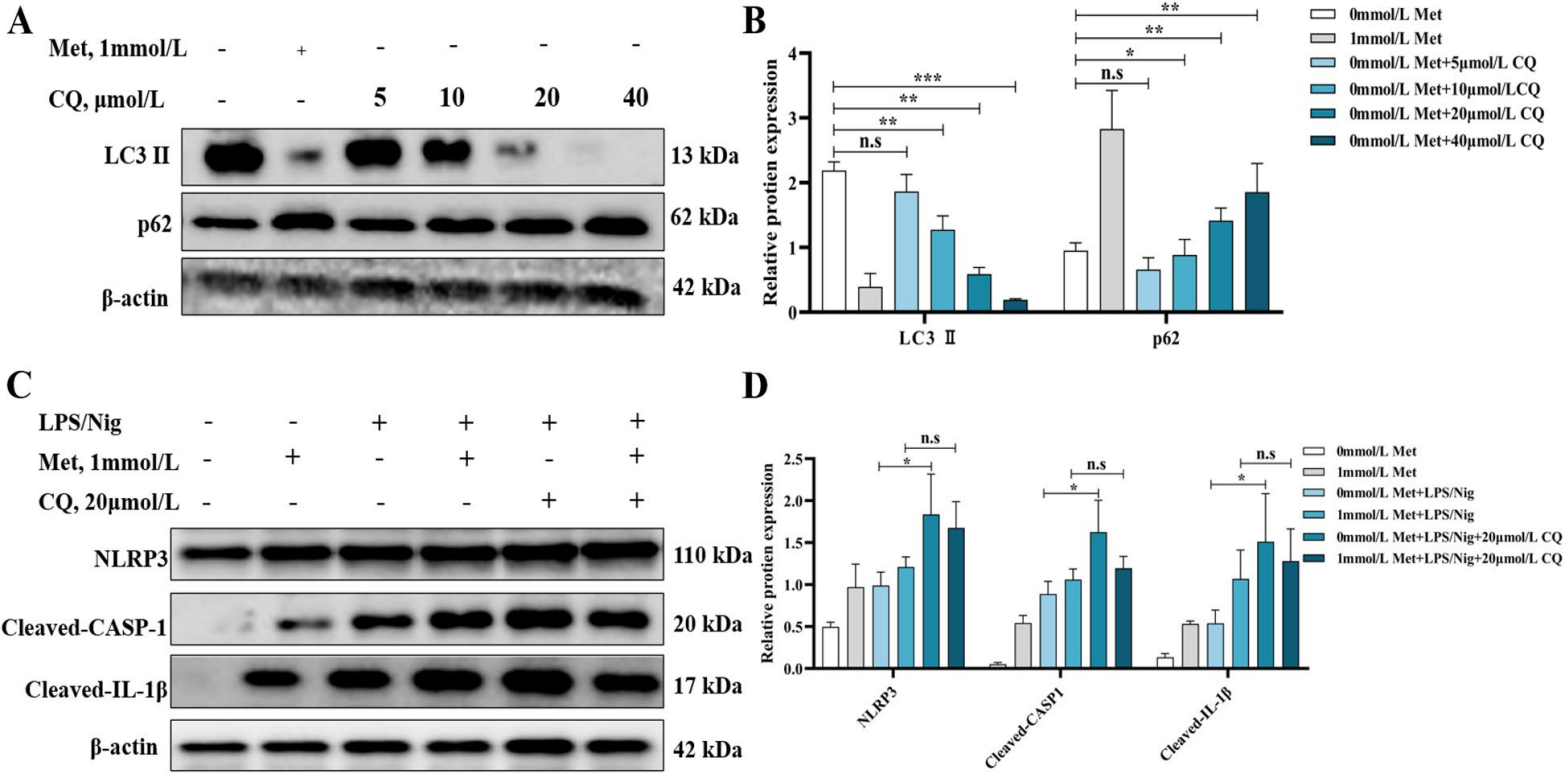
MD inhibited pyroptosis of primary hepatocytes of grass carp through increasing autophagy. Relative protein expression of LC3 II and p62 (**A**) and quantification (**B**) after with or without 1 mmol/L Met and CQ co-treatment for 48 h; relative protein expression of NLRP3, cleaved-caspase-1, cleaved-IL-1β (**C**) and quantification (**D**) after with or without 1 mmol/L Met and 20 μmol/L CQ co-treatment for 48 h followed by 100 ng/mL LPS treatment for 4 h and then 0.5 μmol/L Nig treatment for 0.5 h. The results were expressed as mean ± SD of 3 independent observations. ^*^P < 0.05, ^**^P < 0.01, ^***^P < 0.001, n.s: no significance. Met, methionine; CQ, chloroquine; LC3, microtubule-associated protein 1 light chain 3; p62, sequestosome 1; LPS, lipopolysaccharide; Nig, nigericin sodium salt; NLRP3, NOD-like receptor thermal protein domain associated protein 3; CASP-1, cysteinyl aspartate specific proteinase-1; IL-1β, interleukin-1β

In this experiment, we added 0, 5, 10, 20 or 40 μmol/L CQ to the Met-treated culture medium, and treated the cells for a total of 48 h. It was found that a dose of 20 μmol/L CQ significantly inhibited the protein expression of LC3 II and enhanced the protein expression of p62 (Fig. 6A and B, *P* < 0.05). Then, 20 μmol/L CQ treated cells marked enhanced the expression of NLRP3, cleaved-caspase-1 and cleaved-IL-1β inhibited by MD (Fig. 6C and D, *P* < 0.05).

### MD induced autophagy through activating the ROS-AMPK signaling pathway of primary hepatocytes of grass carp

**Fig. 7:**
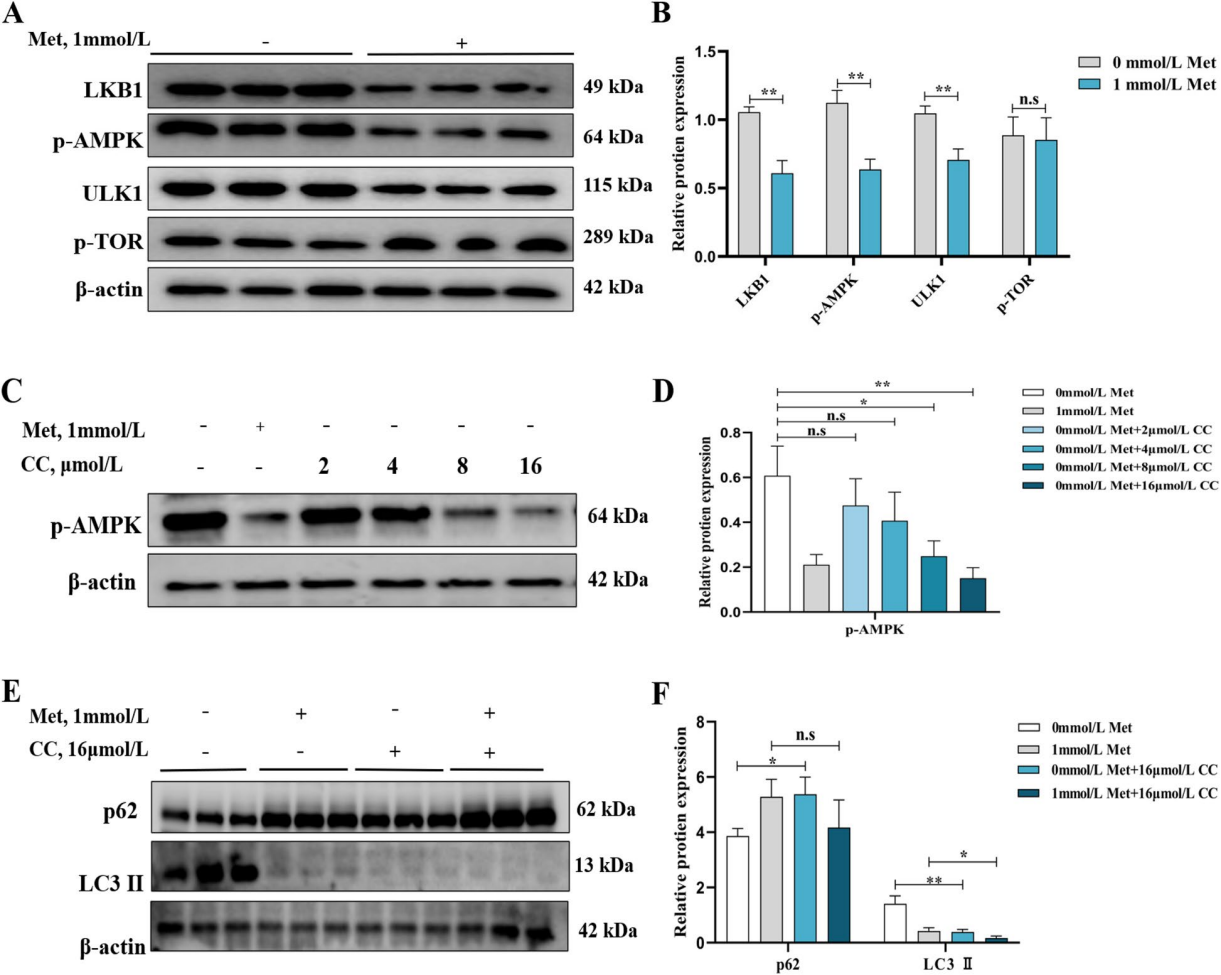
MD promoted autophagy through the AMPK signaling pathway. Relative protein expression of LKB1, p-AMPK, ULK1, p-TOR (**A**) and quantification (**B**) after with or without 1 mmol/L Met treatment for 48 h; relative protein expression of p-AMPK (**C**) and quantification (**D**) after with or without 1 mmol/L Met and different concentrations of CC (0, 2, 4, 8, 16 μmol/L) co-treatment for 48 h; relative protein expression of LC3 II and p62 (**E**) and quantification (**F**) after with or without 1 mmol/L Met and 16 μmol/L CC co-treatment for 48 h. The results were expressed as mean ± SD of 3 independent observations. ^*^P < 0.05, ^**^P < 0.01, n.s: no significance. Met, methionine; LKB1, liver kinase B1; p-AMPK, phosphorylated-AMP-activated protein kinase; ULK1, Unc-51-like kinase 1; p-TOR, phosphorylated-rapamycin; CC, compound C; p62, sequestosome 1; LC3, microtubule-associated protein 1 light chain 3

After treatment with or without 1 mmol/L Met for 48 h, the protein expression of LKB1, p-AMPK and ULK1 were significantly higher (*P* < 0.05), whereas the protein expression of p-TOR changed insignificantly in hepatocytes in the MD group (*P* > 0.05) (Fig. 7A and B). Then we used 0, 2, 4, 8 or 16 μmol/L CC to the Met-treated culture medium for 48 h. This result observed that the protein expression of p-AMPK subsequently decreased with increasing CC dose, significantly at 8 and 16 μmol/L (Fig. 7C and D, *P* < 0.05). Then 16 μmol/L CC treated cells significantly suppressed the level of LC3 II promoted by MD and increased the level of p62 inhibited by MD (Fig. 7E and F, *P* < 0.05).

**Fig. 8:**
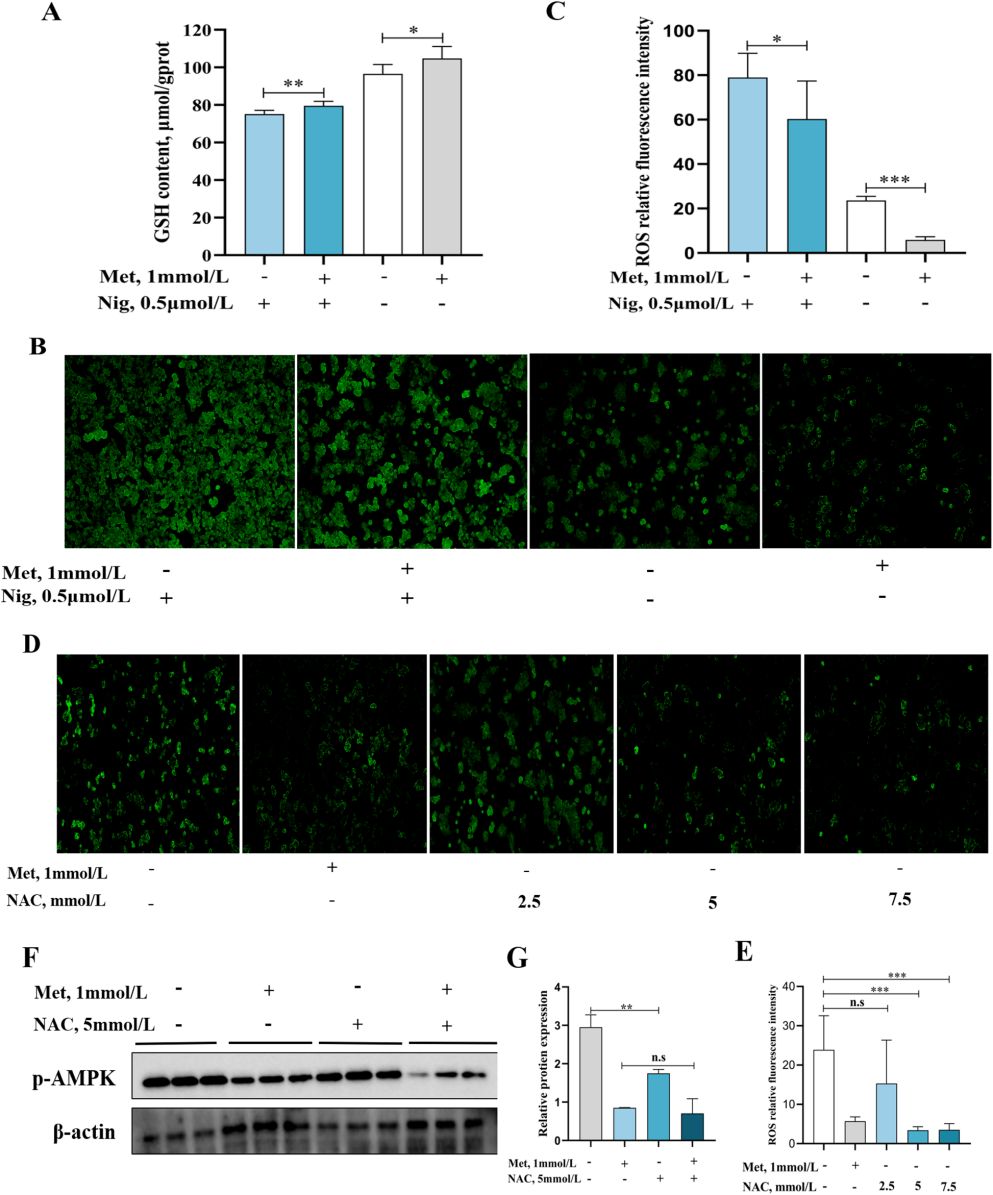
MD activated the AMPK signaling pathway by increasing ROS. Content of GSH (**A**); ROS (**B**) and quantification (**C**) after with or without 1 mmol/L Met treatment for 48 h; content of ROS (**D**) and quantification (**E**) after pre-treatment with NAC (0, 2.5, 5, 7.5 mmol/L) for 1 h then treat with or without 1 mmol/L Met for 48 h; relative protein expression of p-AMPK (**F**) and quantification (**G**) after pre-treatment with 5 mmol/L NAC for 1 h then treat with or without 1 mmol/L Met for 48 h. The results were expressed as mean ± SD of 3 or 6 independent observations (WB: *n* = 3). ^*^P < 0.05, ^**^P < 0.01, ^***^P < 0.001, n.s: no significance. GSH, glutathione; Met, methionine; Nig, nigericin sodium salt; ROS, reactive oxygen species; NAC, *N*-acetyl-L-cysteine; p-AMPK, phosphorylated-AMP-activated protein kinase

In addition, the results of this experiment revealed that in the pyroptosis state, MD significantly reduced GSH content (Fig. 8A, *P* < 0.05), and significantly increased ROS content (Fig. 8B and C, *P* < 0.05). Next, after pretreating the cells with NAC, this result found that the production of ROS was significantly inhibited when the concentration of NAC was 5 mmol/L (Fig. 8D and E, *P* < 0.05). Also, 5 mmol/L NAC pre-treatment significantly inhibited the protein content of AMPK increased by MD (Fig. 8F and G, *P* < 0.05).

## Discussion

### MD increased damage of liver and cell death of primary hepatocytes in grass carp

The liver is the main site of protein synthesis and secretion, and is the center of metabolism in the animals [34]. Histopathological results revealed that the liver of grass carp after MD showed the undesirable phenomena of cell membrane rupture and congestion of hepatic blood sinusoids, resulting in liver injury in grass carp. Similarly, it has been found that MD caused general defective of mitochondria and reduced oxidative capacity in rainbow trout liver [35]. Thus, MD may influence tissue damage.

As mentioned above, excessive cell death leads to tissue damage, so we further examined the effect of MD on cell death in vitro using primary hepatocytes of grass carp. On the first step, we isolated and cultured grass carp primary hepatocytes. The hepatocytes we isolated and cultured were round, with well-defined borders, a nucleus in the center of the cell, and occasionally a binucleate hepatocyte; then two or three cells will join together and begin to grow in sheets; furthermore, the cell membranes of the adjacent cells will fuse with each other; finally, the cells become vacuolated until they died. This conforms to the basic morphological characteristics of hepatocytes, and consistent with previous studies [36–38].

Besides, hepatocytes are the only site of ALB synthesis [39]. After ALB is being synthesized, it would be secreted out of the cell without being stored in the hepatocyte [40]. Therefore, ALB is a functional indicator of hepatocyte function, and its level reflects hepatocyte function [37]. Likewise, in one study, cell identification was accomplished by assaying the expression of ALB in primary hepatocytes of grass carp [41]. In this study, the activity of the cells followed the same trend as the content of ALB in the medium, both reaching their optimal values at 96 h. Therefore, combined with cell morphology and ALB content, we characterized isolated and cultured hepatocytes.

It is widely known that cell viability can directly reflect the proportion of healthy cells [42]. The results found that after treating the cells with different concentrations of Met, the best cell viability was observed at the group with 1 mmol/L Met being companied by the highest amount of ALB in the cell culture medium. Moreover, LDH is present in the cytoplasm of almost all animal tissues and cells, and will be rapidly released from the cells when the cell membrane structure is damaged [43]. Therefore, the activity of LDH can reflect the degree of cellular structure damage. The results of this experiment showed that 1 mmol/L Met resulted in the least amount of LDH released. In addition, it has been shown that after LPS/ATP treatment of RLE-6TN cells, the trend of cell viability was negatively correlated with the activity of LDH in the culture medium [44], which is consistent with the above results.

It is well known that excessive Met can be toxic to animals [45]. Previous studies of our group have shown that either too low or too high dietary Met reduced the growth performance and muscle quality in on-growing grass carp [46]. Excess Met promoted excessive accumulation of ROS in the mouse brain microvascular endothelial cells, which in turn led to increased cell damage [47]. Therefore, the addition of the appropriate Met is conducive to the normal growth of cells as well as the development of the animal organism. In summary, combined with cell viability, ALB content and LDH activity, the optimal concentration of Met for treating cells was 1 mmol/L.

Subsequently, we explored the effects of MD on hepatocyte morphology by TEM, the results found that MD led to cell membrane rupture, increased autophagic vesicle formation, and promoted chromatin condensation in hepatocytes. Similarly, it was found that MD also led to the chromatin margination and nuclear convolution of hepatocytes in *P. fulvidraco* [5]. This further suggests that MD leads to cell death and thus tissue damage. As we described before, the forms of cell death contained apoptosis, pyroptosis, autophagy, necroptosis and so on [15]. So, we next explored the effect of MD on these several modes of cell death.

The Bcl-2 family is divided into two classes, anti-apoptotic Bcl-2 and pro-apoptotic Bax, which are involved in the regulation and amplification of apoptosis [48]. This study found that MD significantly decreased the protein expression of Bcl-2 and increased the protein expression of Bax, which increased the apoptosis. Many previous studies have also found that dietary MD enhanced the protein expression of Bax and reduced the protein expression of Bcl-2 in broiler kidney cells and thymus of the chicken [8, 49]. It has been widely studied that depletion of polyamines may be a generalized factor in the activation of apoptosis [50, 51]. We know that *S*-adenosylmethionine (SAM) generated after Met metabolism involved in the synthesis of polyamines. It has been suggested that Met supplementation can increase polyamine levels in the liver of female C57BL/6 J mice [52]. However, p53 is a tumor suppressor associated with apoptosis and inhibits polyamine biosynthesis [53, 54]. One study reported that MD increased the effect of p53 expression in human embryonic stem cell lines khES3 and 201B7 [55]. Therefore, MD affected apoptosis possibly be related to polyamine synthesis.

During autophagy, both LC3 II and p62 are key protein of autophagy [56]. At the same time, this study found that MD enhanced the protein expression of LC3 II and decreased the protein expression of p62, which increased the autophagy. At present, a few studies found dietary Met-choline deficiency increased autophagy in the liver of male mice [11]. And we know that Met is metabolized in the liver to produce SAM, which acts as an intracellular methyl donor [57]. It has been reported that SAM is also involved in the inhibition of autophagy [58]. Whereas, the methylation of protein phosphatase 2A (PP2A) occurs normally when SAM levels are sufficient, thereby promoting growth and inhibiting autophagy [59, 60]. It has been found that MD leads to a rapid decrease of intracellular SAM content in undifferentiated khES3 cells [52]. The SAM deficiency in the prototrophic CEN.PK strain greatly reduced the protein expression of methylated PP2A [61]. And decreased methylated expression of PP2A increased autophagy in *Drosophila* [62]. Therefore, MD affected autophagy possibly with regard to SAM.

Interestingly, the results showed that MD decreased the protein expression of activated caspase-1, IL-1β, and RIP1. However, at present, no study has been reported on the effect of Met on pyroptosis and necroptosis, which deserves further investigation. Also, the results suggested that the effect of Met on necroptosis may be weak. For this reason, we next focused on and explored the effects of MD on autophagy as well as pyroptosis and its mechanisms.

### MD decreased pyroptosis via enhancing autophagy of primary hepatocytes in grass carp

To explore the above points, we first built a pyroptosis model of primary hepatocyte via specifically activating NLRP3 in grass carp, which acted as a classical inflammasome to activate pyroptosis [63]. These results showed that this model can be successfully established under co-treatment by using 100 ng/mL LPS for 4 h and 0.5 μmol/L Nig for 0.5 h. Previously, several studies have established the pyroptosis model of alveolar macrophages cells using LPS and Nig successfully [64].

Subsequently, on the basis of pyroptosis, the results found that MD further reduced the gene expression of *NLRP3*, *ASC*, *caspase-1*, *GSDME* and *IL-1β* and the protein expression of NLRP3, ASC, cleaved-caspase-1 and cleaved-IL-1β. This suggested that the MD group had a poorer pyroptosis. It has been noted that autophagy levels have a regulatory role in pyroptosis and have been validated and applied in the treatment of tumors, infectious diseases, and cardiovascular and cerebrovascular diseases [65]. Some studies have shown that an increase of autophagy in vulnerable atherosclerotic plaques inhibited the activation of NLRP3, secretion of inflammatory cytokines, thereby alleviating inflammation and attenuating pyroptosis [66]. In addition, some studies have found that increased autophagy reduced protein expression of NLRP3, caspase-1, IL-1β and GSDMD in the injured spinal cord ventral horn grey matter of female C57BL/6 J mice [67]. Similarly, autolysosomes in autophagy inhibited pyroptosis by targeting degradation of inflammasome components (NLRP3) in primary chondrocyte culture of male Sprague–Dawley rats [68]. As mentioned above, the study found that MD can increase autophagy. To verify whether the occurrence of autophagy can affect pyroptosis of primary hepatocytes in grass carp, we used CQ to inhibit the binding of autophagosomes to lysosomes and eventually inhibiting autophagy [69]. The results indicated that the occurrence of autophagy decreased and pyroptosis increased after using 20 μmol/L CQ, which reinforced our results. Consequently, MD inhibited pyroptosis may be associated with triggering autophagy.

### MD induced autophagy via activating ROS-AMPK signaling pathway of primary hepatocytes in grass carp

In addition, the AMPK-TOR signaling pathway has been widely studied as a classical pathway regulating autophagy [13, 70]. It was found that AMPK could directly activate LKB1 and thus activate autophagy; meanwhile, AMPK could further promote autophagy by activating TOR [71]. It turned out that MD markedly increased the protein expression level of LKB1, p-AMPK and ULK1 but had no significant change on p-TOR in hepatocytes. It has been reported that different nutritional status can induce different cellular processes through different upstream signals [72]. One study on *P. fulvidraco* showed that MD or Met excess activated the AMPK and TOR signaling pathways in the liver, respectively [5]. In addition, it was found that MD improved the gene expression of *LKB1* and *AMPK*, but not *TOR*, in the ovarian tissue of female C57BL/6 mice [73]. Furthermore, it was noted that C2C12 cells supplemented with Met for 1 h after nutritional deficiency, increased the expression of TOR proteins [74]. Besides, it has been shown that negative pressure wound therapy can directly induce the beginning of autophagy in rat osteoblasts through the AMPK-ULK1 pathway thereby promoting osteogenic differentiation [75]. This is also consistent with the results of this experiment. Subsequently, we used CC to co-treating cells with Met and found that the protein expression of LC3 II was significantly decreased and p62 was increased in the MD group, which showed that the CC inhibited autophagy in hepatocyte. Thus, MD may activate autophagy mainly by affecting the AMPK signaling pathway.

In addition, AMPK is thought to sense signals associated with changes in mitochondrial dynamics, and since ROS production is largely dependent on mitochondrial activity, ROS production is closely linked to the regulatory role of AMPK [76]. It has been demonstrated that ROS can directly or indirectly activate AMPK, which in turn promotes autophagy [77]. This study has been found that after MD treatment, ROS levels are raised and GSH levels are lowered, which shown that increased oxidative stress in hepatocytes of grass carp. One study has been reported that increased ROS activated the AMPK signaling pathway in mice [78]. Moreover, GSH as a non-enzymatic antioxidant in the body, can scavenge ROS and alleviate oxidative damage [79]. And Met as an important precursor substance of GSH, also has a very important antioxidant role [80]. Similarly, it has been found that dietary MD reduced GSH content in mice [23] and mitochondria of liver in pig [81]. Moreover, free radicals, such as superoxide anion (O^2−^) and hydroxyl radical (OH^−^), are important factors causing oxidative stress [82]. Whereas, previous studies of our group have shown that MD does not properly scavenge O^2−^ and OH^−^, which in turn reduces the antioxidant capacity of grass carp [83]. Thus, MD may lead to oxidative stress in grass carp by reducing the ability of antioxidant enzymes to scavenge free radicals. These results suggested that MD may ultimately activate autophagy by increasing ROS and further activating AMPK. To further verify this, we used the ROS scavenger NAC to pretreat the cells. It was found that the protein expression of p-AMPK was significantly reduced by the use of 5 mmol/L NAC pretreated with cells for 1 h. Thus, MD could activate the expression of AMPK signaling pathway via increasing ROS and finally increase autophagy.

## Conclusions

**Fig. 9:**
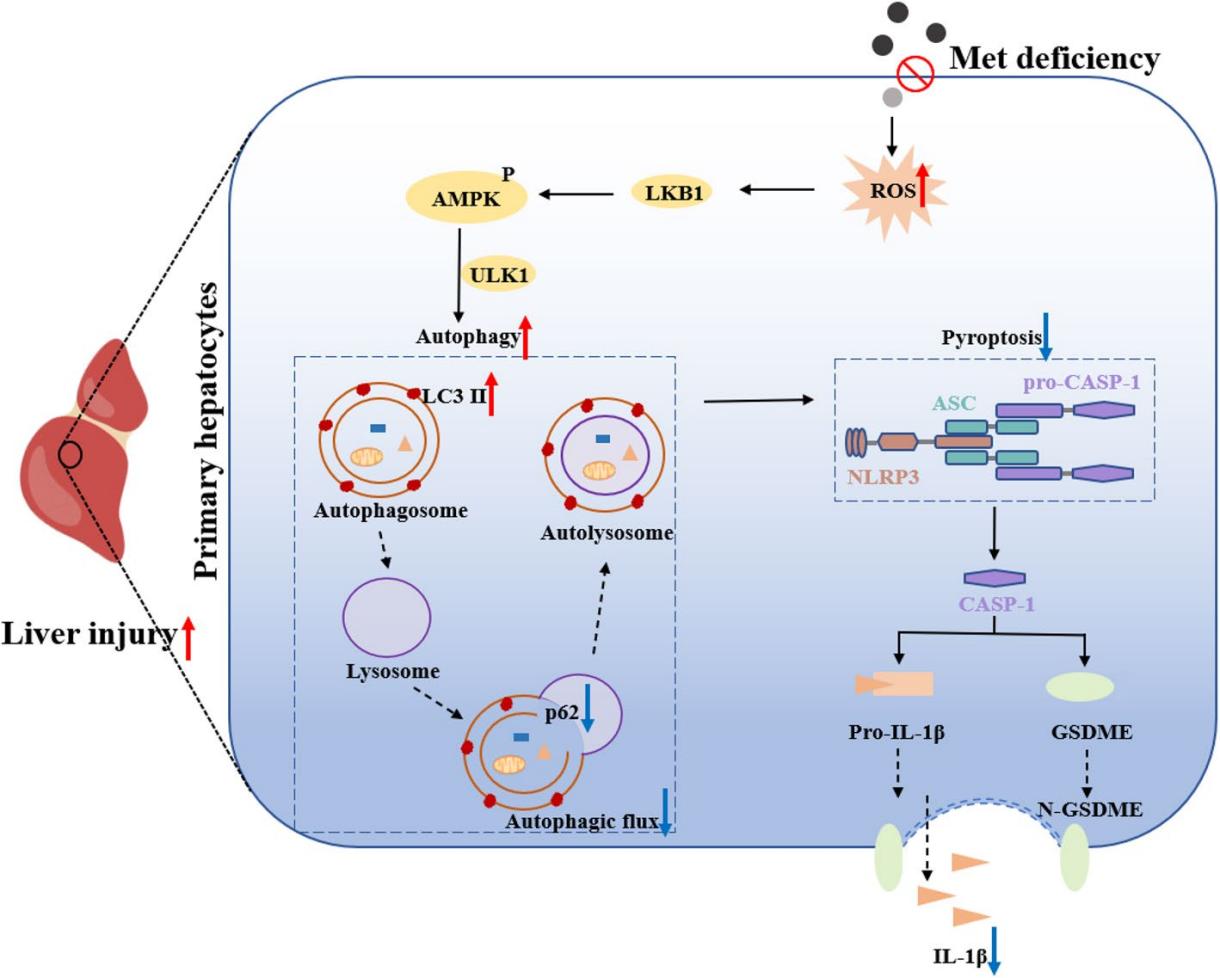
Mechanism of MD induced autophagy but inhibited pyroptosis. MD promoted autophagy to inhibit pyroptosis through the ROS-AMPK signaling pathway of primary hepatocyte in grass carp. ROS, reactive oxygen species; LKB1, liver kinase B1; p-AMPK, phosphorylated-AMP-activated protein kinase; ULK1, Unc-51-like kinase 1; LC3, microtubule-associated protein 1 light chain 3; p62, sequestosome 1; NLRP3, NOD-like receptor thermal protein domain associated protein 3; ASC, apoptosis-associated speck-like protein containing a CARD; CASP-1, cysteinyl aspartate specific proteinase-1; IL-1β, interleukin-1β; GSDME, gasdermin E

In summary, the results of this study suggested that: (1) MD promoted autophagy and apoptosis, but inhibited pyroptosis and necroptosis; (2) MD inhibited pyroptosis may be related regarding the promotion of autophagy; and (3) MD activated AMPK by inducing ROS production which in turn promoted autophagy (Fig. 9). Accordingly, these results suggested the appropriate addition of Met has a potential relationship with liver health.

### Supplementary Information

**Additional file 1:** **Table S1**. Composition and nutrient levels of experimental diets. **Fig.** **S1**. The histology of the of liver of grass carp after different concentrations of Met feeding for 60 d.

## Data Availability

The datasets produced and/or analyzed during the current study are available from the corresponding author on reasonable request.

## Abbreviations

AMPK: Adenosine 5′-monophosphate (AMP)-activated protein kinase
Bax: B-cell lymphoma protein 2
Bcl-2: B-cell lymphoma-2
CC: Compound C
CQ: Chloroquine
IL-1β: Interleukin-1β
LC3: Microtubule-associated protein 1 light chain 3
LKB1: Liver kinase B1
MD: Methionine deficiency
Met: Methionine
NAC: *N*-Acetyl-L-cysteine
p62: Sequestosome 1
RIP1: Receptor-interacting protein kinase 1
RIP3: Receptor-interacting protein kinase 3
ROS: Reactive oxygen species
ULK1: Unc-51-like kinase 1

## References

[1] Khoklang A, Kersanté P, Nontasan S, Sutthi N, Pakdeenarong N, Wang T, et al. Insights into the functional properties of a natural free amino acid mix: effect on growth performance, nutrient metabolism, and immune response in a carnivorous fish, Asian seabass (*Lates calcarifer*). Fish Shellfish Immunol. 2024;144:109232. 10.1016/j.fsi.2023.109232. 37984611 10.1016/j.fsi.2023.109232

[2] Fontagné-Dicharry S, Alami-Durante H, Aragão C, Kaushik S, Geurden I. Parental and early-feeding effects of dietary methionine in rainbow trout (*Oncorhynchus mykiss*). Aquaculture. 2017;469:16–27. 10.1016/j.aquaculture.2016.11.039. 10.1016/j.aquaculture.2016.11.039

[3] Jensen-Cody SO, Potthoff MJ. Hepatokines and metabolism: deciphering communication from the liver. Mol Metab. 2021;44:101138. 10.1016/j.molmet.2020.101138. 33285302 10.1016/j.molmet.2020.101138 PMC7788242

[4] Song BL, Fu M, He F, Zhao H, Wang Y, Nie QH, et al. Methionine deficiency affects liver and kidney health, oxidative stress, and ileum mucosal immunity in broilers. Front Vet Sci. 2021;8:722567. 10.3389/fvets.2021.722567. 34631856 10.3389/fvets.2021.722567 PMC8493001

[5] Song YF, Gao Y, Hogstrand C, Li DD, Pan YX, Luo Z. Upstream regulators of apoptosis mediates methionine-induced changes of lipid metabolism. Cell Signal. 2018;51:176–90. 10.1016/j.cellsig.2018.08.005. 30099089 10.1016/j.cellsig.2018.08.005

[6] Galluzzi L, Vitale I, Aaronson SA, Abrams JM, Adam D, Agostinis P, et al. Molecular mechanisms of cell death: recommendations of the nomenclature committee on cell death 2018. Cell Death Differ. 2018;25(3):486–541. 10.1038/s41418-017-0012-4. 29362479 10.1038/s41418-017-0012-4 PMC5864239

[7] Green DR. The coming decade of cell death research: five riddles. Cell. 2019;177(5):1094–107. 10.1016/j.cell.2019.04.024. 31100266 10.1016/j.cell.2019.04.024 PMC6534278

[8] Song BL, Zeng QM, Liu Y, Wu BY. Effect of methionine deficiency on the apoptosis and cell cycle of kidney in broilers. Res Vet Sci. 2019;135:228–36. 10.1016/j.rvsc.2019.09.013. 31648780 10.1016/j.rvsc.2019.09.013

[9] Glick D, Barth S, Macleod KF. Autophagy: cellular and molecular mechanisms. J Pathol. 2010;221(1):3–12. 10.1002/path.2697. 20225336 10.1002/path.2697 PMC2990190

[10] Li XH, He SK, Ma BY. Autophagy and autophagy-related proteins in cancer. Mol Cancer. 2020;19(1):12. 10.1186/s12943-020-1138-4. 31969156 10.1186/s12943-020-1138-4 PMC6975070

[11] Veskovic M, Mladenovic D, Milenkovic M, Tosic J, Borozan S, Gopcevic K, et al. Betaine modulates oxidative stress, inflammation, apoptosis, autophagy, and Akt/mTOR signaling in methionine-choline deficiency-induced fatty liver disease. Eur J Pharmacol. 2019;848:39–48. 10.1016/j.ejphar.2019.01.043. 30689995 10.1016/j.ejphar.2019.01.043

[12] Zhou Y, Zhou Z, Peng J, Loor JJ. Methionine and valine activate the mammalian target of rapamycin complex 1 pathway through heterodimeric amino acid taste receptor (TAS1R1/TAS1R3) and intracellular Ca^2+^ in bovine mammary epithelial cells. J Dairy Sci. 2018;101(12):11354–63. 10.3168/jds.2018-14461. 30268610 10.3168/jds.2018-14461

[13] Kim J, Kundu M, Viollet B, Guan KL. AMPK and mTOR regulate autophagy through direct phosphorylation of ULK1. Nat Cell Biol. 2011;13(2):132–41. 10.1038/ncb2152. 21258367 10.1038/ncb2152 PMC3987946

[14] Ji J, Xu YB, Zheng MZ, Luo CL, Lei HT, Qu H, et al. Methionine attenuates lipopolysaccharide-induced inflammatory responses via DNA methylation in macrophages. ACS Omega. 2019;4(1):2331–6. 10.1021/acsomega.8b03571. 30775649 10.1021/acsomega.8b03571 PMC6374979

[15] Moujalled D, Strasser A, Liddell JR. Molecular mechanisms of cell death in neurological diseases. Cell Death Differ. 2021;28(7):2029–44. 10.1038/s41418-021-00814-y. 34099897 10.1038/s41418-021-00814-y PMC8257776

[16] Man SM, Karki R, Kanneganti TD. Molecular mechanisms and functions of pyroptosis, inflammatory caspases and inflammasomes in infectious diseases. Immunol Rev. 2017;277(1):61–75. 10.1111/imr.12534. 28462526 10.1111/imr.12534 PMC5416822

[17] Opdenbosch N, Lamkanfi M. Caspases in cell death, inflammation, and disease. Immunity. 2019;50(6):1352–64. 10.1016/j.immuni.2019.05.020. 31216460 10.1016/j.immuni.2019.05.020 PMC6611727

[18] Shi JJ, Gao WQ, Shao F. Pyroptosis: gasdermin-mediated programmed necrotic cell death. Trends Biochem Sci. 2017;42(4):245–54. 10.1016/j.tibs.2016.10.004. 27932073 10.1016/j.tibs.2016.10.004

[19] Forn-Cuní G, Meijer AH, Varela M. Zebrafish in inflammasome research. Cells. 2019;8(8):901. 10.3390/cells8080901. 31443239 10.3390/cells8080901 PMC6721725

[20] Morimoto N, Kono T, Sakai M, Hikima JI. Inflammasomes in teleosts: structures and mechanisms that induce pyroptosis during bacterial infection. Int J Mol Sci. 2021;22(9):4389. 10.3390/ijms22094389. 33922312 10.3390/ijms22094389 PMC8122782

[21] Song ZX, Zou JH, Wang MY, Chen ZW, Wang QC. A comparative review of pyroptosis in mammals and fish. J Inflamm Res. 2022;15:2323–31. 10.3390/ijms22094389. 35431566 10.3390/ijms22094389 PMC9012342

[22] Wang ZX, Liang MC, Li H, Cai L, He HJ, Wu Q, et al. L-Methionine activates Nrf2-ARE pathway to induce endogenous antioxidant activity for depressing ROS-derived oxidative stress in growing rats. J Sci Food Agric. 2019;99(10):4849–62. 10.1002/jsfa.9757. 31001831 10.1002/jsfa.9757

[23] Wanders D, Stone KP, Forney LA, Cortez CC, Dille KN, Simon J, et al. Role of GCN2-independent signaling through a noncanonical PERK/NRF2 pathway in the physiological responses to dietary methionine restriction. Diabetes. 2016;65(6):1499–510. 10.2337/db15-1324. 26936965 10.2337/db15-1324 PMC4878423

[24] Zhao J, Liu ZY, Chang ZH. Lipopolysaccharide induces vascular endothelial cell pyroptosis via the SP1/RCN2/ROS signaling pathway. Eur J Cell Biol. 2021;100(4):151164. 10.1016/j.ejcb.2021.151164. 34004559 10.1016/j.ejcb.2021.151164

[25] Cheng YC, Chu LW, Chen JY, Hsieh SL, Chang YC, Dai ZK, et al. Loganin attenuates high glucose-induced Schwann cells pyroptosis by inhibiting ROS generation and NLRP3 inflammasome activation. Cells. 2020;9(9):1948. 10.3390/cells9091948. 32842536 10.3390/cells9091948 PMC7564733

[26] FAO. The state of world fisheries and aquaculture 2024-blue transformation in action. FAO; 2024. https://openknowledge.fao.org/handle/20.500.14283/cd0690zh.

[27] Fang CC, Feng L, Jiang WD, Wu P, Liu Y, Kuang SY, et al. Effects of dietary methionine on growth performance, muscle nutritive deposition, muscle fibre growth and type I collagen synthesis of on-growing grass carp (*Ctenopharyngodon idella*). Br J Nutr. 2021;126(3):321–36. 10.1017/S0007114520002998. 32718370 10.1017/S0007114520002998

[28] Shi YY, Ma J, Wang ZJ, Ye YT, Jiang R, Kong QW. Protective effects of three natural plants water soluble matter on primary hepatocyte of grass carp (*Ctenopharyngodon idellus*) damaged by hydrogen peroxide. Aquaculture. 2022;43(10):48–56. 10.13302/j.cnki.fi.2022.10.008. 10.13302/j.cnki.fi.2022.10.008

[29] Rakesh KJ, Jaswinder SM, Shiv KS. Albumin in advanced liver diseases: the good and bad of a drug. Hepatology. 2021;74(5):2848–62. 10.1002/hep.31836. 33772846 10.1002/hep.31836

[30] Wu P, Jiang J, Liu Y, Hu K, Jiang WD, Li SH, et al. Dietary choline modulates immune responses, and gene expressions of TOR and eIF4E-binding protein2 in immune organs of juvenile Jian carp (*Cyprinus carpio* var. Jian). Fish Shellfish Immunol. 2013;35(3):697–706. 10.1016/j.fsi.2013.05.030. 23774323 10.1016/j.fsi.2013.05.030

[31] Su JG, Zhang RF, Dong J, Yang CR. Evaluation of internal control genes for qRT-PCR normalization in tissues and cell culture for antiviral studies of grass carp (*Ctenopharyngodon idella*). Fish Shellfish Immunol. 2011;30:830–5. 10.1016/j.fsi.2011.01.006. 21255653 10.1016/j.fsi.2011.01.006

[32] Zhao FZ, Maren NA, Kosentka PZ, Liao YY, Lu HY, Duduit JR, et al. An optimized protocol for stepwise optimization of real-time RT-PCR analysis. Hortic Res. 2021;8(1):179. 10.1038/s41438-021-00616-w. 34333545 10.1038/s41438-021-00616-w PMC8325682

[33] Yang PC, Mahmood T. Western blot: technique, theory, and trouble shooting. North Am J Med Sci. 2012;4(9):429–34. 10.4103/1947-2714.100998. 10.4103/1947-2714.100998 PMC3456489 23050259

[34] Amouzandeh M, Sundström A, Wahlin S, Wernerman J, Rooyackers O, Norberg Å. Albumin and fibrinogen synthesis rates in advanced chronic liver disease. Am J Physiol Gastrointest Liver Physiol. 2023;325(5):G391–7. 10.1152/ajpgi.00072.2023. 37605837 10.1152/ajpgi.00072.2023

[35] Séité S, Mourier A, Camougrand N, Salin B, Figueiredo-Silva AC, Fontagné-Dicharry S, et al. Dietary methionine deficiency affects oxidative status, mitochondrial integrity and mitophagy in the liver of rainbow trout (*Oncorhynchus mykiss*). Sci Rep. 2018;8:10151. 10.1038/s41598-018-28559-8. 29977029 10.1038/s41598-018-28559-8 PMC6033930

[36] Qin J, Ye YT, Leng XJ, Cai CF, Song L, Xu F, et al. Isolation and primary culture of hepatocytes from *Ctenopharyngodon idella*. Acta Lab Anim Sci Sin. 2012;20(3):33–40. 10.3969/j.issn.1005-4847.2012.03.008. 10.3969/j.issn.1005-4847.2012.03.008

[37] Charni-Natan M, Goldstein I. Protocol for primary mouse hepatocyte isolation. STAR Protoc. 2020;1(2):100086. 10.1016/j.xpro.2020.100086. 33111119 10.1016/j.xpro.2020.100086 PMC7580103

[38] Jiang QD, Li HP, Liu FJ, Wang XJ, Guo YJ, Wang LF, et al. Isolation and identification of bovine primary hepatocytes. Genet Mol Res. 2013;12(4):5186–94. 10.4238/2013.October.30.3. 24301779 10.4238/2013.October.30.3

[39] Ruot B, Breuillé D, Rambourdin F, Bayle G, Capitan P, Obled C. Synthesis rate of plasma albumin is a good indicator of liver albumin synthesis in sepsis. Am J Physiol Endocrinol Metab. 2000;279(2):E244–251. 10.1152/ajpendo.2000.279.2.E244. 10913022 10.1152/ajpendo.2000.279.2.E244

[40] Spinella R, Sawhney R, Jalan R. Albumin in chronic liver disease: structure, functions and therapeutic implications. Hepatol Int. 2016;10(1):124–32. 10.1007/s12072-015-9665-6. 26420218 10.1007/s12072-015-9665-6

[41] Qin J. Study on the mechanism of hepatocellular injury of grass carp (*Ctenopharyngodon idellus*) by oxidized fats and oils. Shanghai: Shanghai Ocean University; 2012.

[42] Präbst K, Engelhardt H, Ringgeler S, Hübner H. Basic colorimetric proliferation assays: MTT, WST, and resazurin. Methods Mol Biol. 2017;1601:1–17. 10.1007/978-1-4939-6960-9_1. 28470513 10.1007/978-1-4939-6960-9_1

[43] Claps G, Faouzi S, Quidville V, Chehade F, Shen SS, Vagner S, et al. The multiple roles of LDH in cancer. Nat Rev Clin Oncol. 2022;19(12):749–62. 10.1038/s41571-022-00686-2. 36207413 10.1038/s41571-022-00686-2

[44] Zhou T, Li Z, Chen H. Melatonin alleviates lipopolysaccharide (LPS)/adenosine triphosphate (ATP)-induced pyroptosis in rat alveolar type II cells (RLE-6TN) through nuclear factor erythroid 2-related factor 2 (Nrf2)-driven reactive oxygen species (ROS) downregulation. Bioengineered. 2022;13(1):1880–92. 10.1080/21655979.2021.2018981. 35109747 10.1080/21655979.2021.2018981 PMC8973817

[45] Garlick PJ. Toxicity of methionine in humans. J Nutr. 2006;136(6):1722S–1725S. 10.1093/jn/136.6.1722S. 16702346 10.1093/jn/136.6.1722S

[46] Fang CC. Effects and mechanisms of different levels of methionine on growth performance and flesh quality as well as mechanism in on-growing grass carp (*Ctenopharyngodon idella*). Sichuan: Sichuan Agricultural University; 2020.

[47] Tyagi N, Moshal KS, Sen U, Vacek TP, Kumar M, Hughes WM, et al. H2S protects against methionine-induced oxidative stress in brain endothelial cells. Antioxid Redox Signal. 2009;11(1):25–33. 10.1089/ars.2008.2073. 18837652 10.1089/ars.2008.2073 PMC2742910

[48] Kashyap D, Garg VK, Goel N. Intrinsic and extrinsic pathways of apoptosis: role in cancer development and prognosis. Adv Protein Chem Struct Biol. 2021;125:73–120. 10.1016/bs.apcsb.2021.01.003. 33931145 10.1016/bs.apcsb.2021.01.003

[49] Wu B, Chen SS. Effect of methionine deficiency on the thymus and the subsets and proliferation of peripheral blood T-cell, and serum IL-2 contents in broilers. J Integr Agric. 2012;11(6):1009–19. 10.1016/S2095-3119(12)60093-8

[50] Sanderson SM, Gao X, Dai Z, Locasale JW. Methionine metabolism in health and cancer: a nexus of diet and precision medicine. Nat Rev Cancer. 2019;19(11):625–37. 10.1038/s41568-019-0187-8. 31515518 10.1038/s41568-019-0187-8

[51] Seiler N, Raul F. Polyamines and apoptosis. J Cell Mol Med. 2005;9(3):623–42. 10.1111/j.1582-4934.2005.tb00493.x. 16202210 10.1111/j.1582-4934.2005.tb00493.x PMC6741638

[52] Correa-Fiz F, Reyes-Palomares A, Fajardo I, Melgarejo E, Gutiérrez A, García-Ranea JA, et al. Regulatory cross-talk of mouse liver polyamine and methionine metabolic pathways: a systemic approach to its physio pathological consequences. Amino Acids. 2012;42(2–3):577–95. 10.1007/s00726-011-1044-6. 21818563 10.1007/s00726-011-1044-6

[53] Li L, Mao YX, Zhao LN, Li LJ, Wu JJ, Zhao MJ, et al. P53 regulation of ammonia metabolism through urea cycle controls polyamine biosynthesis. Nature. 2019;567(7747):253–6. 10.1038/s41586-019-0996-7. 30842655 10.1038/s41586-019-0996-7

[54] Zhao YH, Chen YX, Wei L, Ran JH, Wang KJ, Zhu SJ, et al. P53 inhibits the urea cycle and represses polyamine biosynthesis in glioma cell lines. Metab Brain Dis. 2023;38(4):1143–53. 10.1007/s11011-023-01173-y. 36745250 10.1007/s11011-023-01173-y

[55] Shiraki N, Shiraki Y, Tsuyama T, Obata F, Miura M, Nagae G, et al. Methionine metabolism regulates maintenance and differentiation of human pluripotent stem cells. Cell Metab. 2014;19(5):780–94. 10.1016/j.cmet.2014.03.017. 24746804 10.1016/j.cmet.2014.03.017

[56] Klionsky DJ, Petroni G, Amaravadi RK, Baehrecke EH, Ballabio A, Boya P, et al. Autophagy in major human diseases. EMBO J. 2021;40(19):e108863. 10.15252/embj.2021108863. 34459017 10.15252/embj.2021108863 PMC8488577

[57] Elango R. Methionine nutrition and metabolism: insights from animal studies to inform human nutrition. J Nutr. 2020;150(Suppl 1):2518S–2523S. 10.1093/jn/nxaa155. 33000159 10.1093/jn/nxaa155

[58] Ouyang Y, Wu Q, Li JJ, Sun S, Sun SR. S-adenosylmethionine: a metabolite critical to the regulation of autophagy. Cell Prolif. 2020;53(11):e12891. 10.1111/cpr.12891. 33030764 10.1111/cpr.12891 PMC7653241

[59] Leulliot N, Quevillon-Cheruel S, Sorel I, De La Sierra-Gallay IL, Collinet B, Graille M, et al. Structure of protein phosphatase methyltransferase 1 (PPM1), a leucine carboxyl methyltransferase involved in the regulation of protein phosphatase 2A activity. J Biol Chem. 2004;279(9):8351–8. 10.1074/jbc.M311484200. 14660564 10.1074/jbc.M311484200

[60] Kitada M, Ogura Y, Monno I, Xu J, Koya D. Effect of methionine restriction on aging: its relationship to oxidative stress. Biomedicines. 2021;9(2):130. 10.3390/biomedicines9020130. 33572965 10.3390/biomedicines9020130 PMC7911310

[61] Sutter BM, Wu X, Laxman S, Tu BP. Methionine inhibits autophagy and promotes growth by inducing the SAM-responsive methylation of PP2A. Cell. 2013;154(2):403–15. 10.1016/j.cell.2013.06.041. 23870128 10.1016/j.cell.2013.06.041 PMC3774293

[62] Bánréti Á, Lukácsovich T, Csikós G, Erdélyi M, Sass M. PP2A regulates autophagy in two alternative ways in *Drosophila*. Autophagy. 2012;8(4):623–36. 10.4161/auto.19081. 22330894 10.4161/auto.19081

[63] Vande Walle L, Lamkanfi M. Pyroptosis. Curr Biol CB. 2016;26:R568–72. 10.1016/j.cub.2016.02.019. 27404251 10.1016/j.cub.2016.02.019

[64] Zou L, Yu Q, Zhang L, Yuan X, Fang F, Xu F. Identification of inflammation related lncRNAs and Gm33647 as a potential regulator in septic acute lung injury. Life Sci. 2021;282:119814. 10.1016/j.lfs.2021.119814. 34298039 10.1016/j.lfs.2021.119814

[65] Pang Q, Wang P, Pan Y, Dong X, Zhou T, Song X, et al. Irisin protects against vascular calcification by activating autophagy and inhibiting NLRP3-mediated vascular smooth muscle cell pyroptosis in chronic kidney disease. Cell Death Dis. 2022;13(3):283. 10.1038/s41419-022-04735-7. 35354793 10.1038/s41419-022-04735-7 PMC8967887

[66] Peng S, Xu LW, Che XY, Xiao QQ, Pu J, Shao Q, et al. Atorvastatin inhibits inflammatory response, attenuates lipid deposition, and improves the stability of vulnerable atherosclerotic plaques by modulating autophagy. Front Pharmacol. 2018;9:438. 10.3389/fphar.2018.00438. 29773990 10.3389/fphar.2018.00438 PMC5943597

[67] Zhang H, Ni W, Yu G, Geng Y, Lou J, Qi J, et al. 3,4-Dimethoxychalcone, a caloric restriction mimetic, enhances TFEB-mediated autophagy and alleviates pyroptosis and necroptosis after spinal cord injury. Theranostics. 2023;13(2):810–32. 10.7150/thno.78370. 36632211 10.7150/thno.78370 PMC9830432

[68] Li Z, Huang Z, Zhang H, Lu J, Tian Y, Piao S, et al. Moderate-intensity exercise alleviates pyroptosis by promoting autophagy in osteoarthritis via the P2X7/AMPK/mTOR axis. Cell Death Discov. 2021;7(1):346. 10.1038/s41420-021-00746-z. 34759265 10.1038/s41420-021-00746-z PMC8580998

[69] Kimura T, Takabatake Y, Takahashi A, Isaka Y. Chloroquine in cancer therapy: a double-edged sword of autophagy. Cancer Res. 2013;73(1):3–7. 10.1158/0008-5472.CAN-12-2464. 23288916 10.1158/0008-5472.CAN-12-2464

[70] Kim YC, Guan KL. mTOR: a pharmacologic target for autophagy regulation. J Clin Invest. 2015;125(1):25–32. 10.1172/JCI73939. 25654547 10.1172/JCI73939 PMC4382265

[71] Herzig S, Shaw RJ. AMPK: guardian of metabolism and mitochondrial homeostasis. Nat Rev Mol Cell Biol. 2018;19(2):121–35. 10.1038/nrm.2017.95. 28974774 10.1038/nrm.2017.95 PMC5780224

[72] Yen CL, Mar MH, Craciunescu CN, Edwards LJ, Zeisel SH. Deficiency in methionine, tryptophan, isoleucine, or choline induces apoptosis in cultured cells. J Nutr. 2002;132(7):1840–7. 10.1093/jn/132.7.1840. 12097657 10.1093/jn/132.7.1840

[73] Harphoush S, Wu G, Zaitoun M, Shi Y, Le G. Methionine restriction diet improves metabolic function in obese C57BL/6 female mice via AMPK/SIRT1/PGC1α pathway. J Food Nutr Res. 2019;7(2):96–104.

[74] Zhou YF, Ren J, Song TX, Peng J, Wei HK. Methionine regulates mTORC1 via the T1R1/T1R3-PLCβ-Ca^2+^-ERK1/2 signal transduction process in C2C12 cells. Int J Mol Sci. 2016;17(10):1684. 10.3390/ijms17101684. 27727170 10.3390/ijms17101684 PMC5085716

[75] Zhang S, Xie Y, Yan F, Zhang Y, Yang Z, Chen Z, et al. Negative pressure wound therapy improves bone regeneration by promoting osteogenic differentiation via the AMPK-ULK1-autophagy axis. Autophagy. 2022;18(9):2229–45. 10.1080/15548627.2021.2016231. 34964701 10.1080/15548627.2021.2016231 PMC9466622

[76] Agostini F, Bisaglia M, Plotegher N. Linking ROS levels to autophagy: the key role of AMPK. Antioxidants. 2023;12(7):1406. 10.3390/antiox12071406. 37507945 10.3390/antiox12071406 PMC10376219

[77] Sun Q, Zhen P, Li DD, Liu XC, Ding XL, Liu HH. Amentoflavone promotes ferroptosis by regulating reactive oxygen species (ROS)/5’AMP-activated protein kinase (AMPK)/mammalian target of rapamycin (mTOR) to inhibit the malignant progression of endometrial carcinoma cells. Bioengineered. 2022;13(5):13269–79. 10.1080/21655979.2022.2079256. 35635082 10.1080/21655979.2022.2079256 PMC9275900

[78] Guo HR, Ouyang YJ, Yin H, Cui HM, Deng HD, Liu H, et al. Induction of autophagy via the ROS-dependent AMPK-mTOR pathway protects copper-induced spermatogenesis disorder. Redox Biol. 2022;49:102227. 10.1016/j.redox.2021.102227. 34979450 10.1016/j.redox.2021.102227 PMC8728583

[79] Liu T, Sun L, Zhang Y, Wang Y, Zheng J. Imbalanced GSH/ROS and sequential cell death. J Biochem Mol Toxicol. 2022;36(1):e22942. 10.1002/jbt.22942. 34725879 10.1002/jbt.22942

[80] Levine RL, Moskovitz J, Stadtman ER. Oxidation of methionine in proteins: roles in antioxidant defense and cellular regulation. IUBMB Life. 2000;50(4–5):301–7. 10.1080/713803735. 11327324 10.1080/713803735

[81] Ying Y, Yun J, Wu GY, Sun KJ, Dai ZL, Wu ZL. Dietary L-methionine restriction decreases oxidative stress in porcine liver mitochondria. Exp Gerontol. 2015;65:35–41. 10.1016/j.exger.2015.03.004. 25765145 10.1016/j.exger.2015.03.004

[82] Martemucci G, Costagliola C, Mariano M, D’andrea L, Napolitano P, D’Alessandro AG. Free radical properties, source and targets, antioxidant consumption and health. Oxygen. 2022;2(2):48–78. 10.3390/oxygen2020006

[83] Tang BR. Effects of dietary methionine on digestive and absorptive ability and antioxidative ability of young grass carp (*Ctenopharyngodon idell*). Sichuan: Sichuan Agricultural University; 2012.

