# Supplementary material for: Methionine deficiency inhibited pyroptosis in primary hepatocytes of grass carp (Ctenopharyngodon idella): possibly via activating the ROS-AMPK-autophagy axis
Source: J Anim Sci Biotechnol. 2024 Sep 2;15:116. doi: 10.1186/s40104-024-01069-6 (PMC11368015; doi:10.1186/s40104-024-01069-6)
Supplement: Supplementary file 1 — Additional file 1: Table S1. Composition and nutrient levels of experimental diets. Fig. S1. The histology of the of liver of grass carp after different concentrations of Met feeding for 60 d. [file 40104_2024_1069_MOESM1_ESM.docx]

**Table S1** Composition and nutrient levels of experimental diets

| **Item** | **Met levels, g/kg diet** | | | | | |
| --- | --- | --- | --- | --- | --- | --- |
|  | **2.54** | **4.85** | **7.43** | **10.12** | **12.40** | **15.11** |
| Ingredients, % |  |  |  |  |  |  |
| Fish meal | 6.80 | 6.80 | 6.80 | 6.80 | 6.80 | 6.80 |
| Gelatin | 4.00 | 4.00 | 4.00 | 4.00 | 4.00 | 4.00 |
| Soybean protein concentrate | 11.00 | 11.00 | 11.00 | 11.00 | 11.00 | 11.00 |
| Crystal amino acid mix ^a^ | 14.28 | 14.28 | 14.28 | 14.28 | 14.28 | 14.28 |
| DL-Methionine (97.7%) | 0.00 | 0.26 | 0.51 | 0.77 | 1.02 | 1.28 |
| L-Glycine (99.0%) | 0.64 | 0.51 | 0.38 | 0.25 | 0.13 | 0.00 |
| α-Starch | 22.00 | 22.00 | 22.00 | 22.00 | 22.00 | 22.00 |
| Corn starch | 26.55 | 26.43 | 26.30 | 26.17 | 26.04 | 25.91 |
| Cellulose | 5.00 | 5.00 | 5.00 | 5.00 | 5.00 | 5.00 |
| Fish oil | 2.48 | 2.48 | 2.48 | 2.48 | 2.48 | 2.48 |
| Soybean oil | 1.80 | 1.80 | 1.80 | 1.80 | 1.80 | 1.80 |
| Ca(H_2_PO_4_)_2_ | 1.40 | 1.40 | 1.40 | 1.40 | 1.40 | 1.40 |
| Vitamin premix^b^ | 2.00 | 2.00 | 2.00 | 2.00 | 2.00 | 2.00 |
| Mineral premix^c^ | 1.00 | 1.00 | 1.00 | 1.00 | 1.00 | 1.00 |
| Choline chloride (50%)^c^ | 1.00 | 1.00 | 1.00 | 1.00 | 1.00 | 1.00 |
| Ethoxyquin (30%) | 0.05 | 0.05 | 0.05 | 0.05 | 0.05 | 0.05 |
| Total | 100.00 | 100.00 | 100.00 | 100.00 | 100.00 | 100.00 |
| Nutrient levels, % |  |  |  |  |  |  |
| Crude protein^d^ | 28.74 | 28.52 | 28.84 | 28.78 | 28.56 | 28.86 |
| Crude lipid^d^ | 4.82 | 4.75 | 4.95 | 4.66 | 4.76 | 4.92 |
| n-3^e^ | 1.04 | 1.04 | 1.04 | 1.04 | 1.04 | 1.04 |
| n-6^e^ | 0.96 | 0.96 | 0.96 | 0.96 | 0.96 | 0.96 |
| Available phosphorus^f^ | 0.40 | 0.40 | 0.40 | 0.40 | 0.40 | 0.40 |
| Methionine | 0.25 | 0.49 | 0.74 | 1.01 | 1.24 | 1.51 |
| Cysteine | 0.16 | 0.15 | 0.16 | 0.15 | 0.15 | 0.15 |

^a^ Crystal amino acid mix (g/kg diet): arginine, 1.86; histidine, 4.90; isoleucine, 5.29; leucine, 1.92; lysine, 5.12; phenylalanine, 3.64; threonine, 6.53; tryptophan, 2.17; valine, 7.00; tyrosine, 4.11; glutamic acid, 54.12; glycine, 48.15, respectively

^b^ Per kilogram of vitamin premix (g/kg): retinyl acetate (1,000,000 IU/g), 0.19; cholecalciferol (500,000 IU/g), 0.20; DL-a-tocopherol acetate (50%), 23.23; menadione (96%), 1.98; cyanocobalamin (1%), 0.94; D-biotin (2%), 0.75; folic acid (95%), 0.17; thiamine nitrate (98%), 0.09; acrophyll acetate (95%), 9.77; niacin (99%), 3.44; meso-inositol (97%), 28.53; calcium-D-pantothenate (90%), 4.19; riboflavin (80%), 0.73; pyridoxine hydrochloride (98%), 0.45. All ingredients were diluted with corn starch to 1 kg

^c^ Per kilogram of mineral premix (g/kg): MnSO_4_·H_2_O (31.8% Mn), 2.6590; MgSO_4_·H_2_O (15.0% Mg), 256.7933; FeSO_4_·H_2_O (30.0% Fe), 12.6083; ZnSO_4_·H_2_O (34.5% Zn), 8.8700; CuSO_4_·5H_2_O (25.0% Cu), 0.9560; CaI_2_ (3.2% I), 1.5625; Na_2_SeO_3_ (44.7% Se), 0.0611. All ingredients were diluted with corn starch to 1 kg

^d^ Crude protein and lipid contents were measured values

^e^ n-3 and n-6 were calculated according to NRC (2011)

^f^ Available phosphorus was calculated according to NRC (2011)


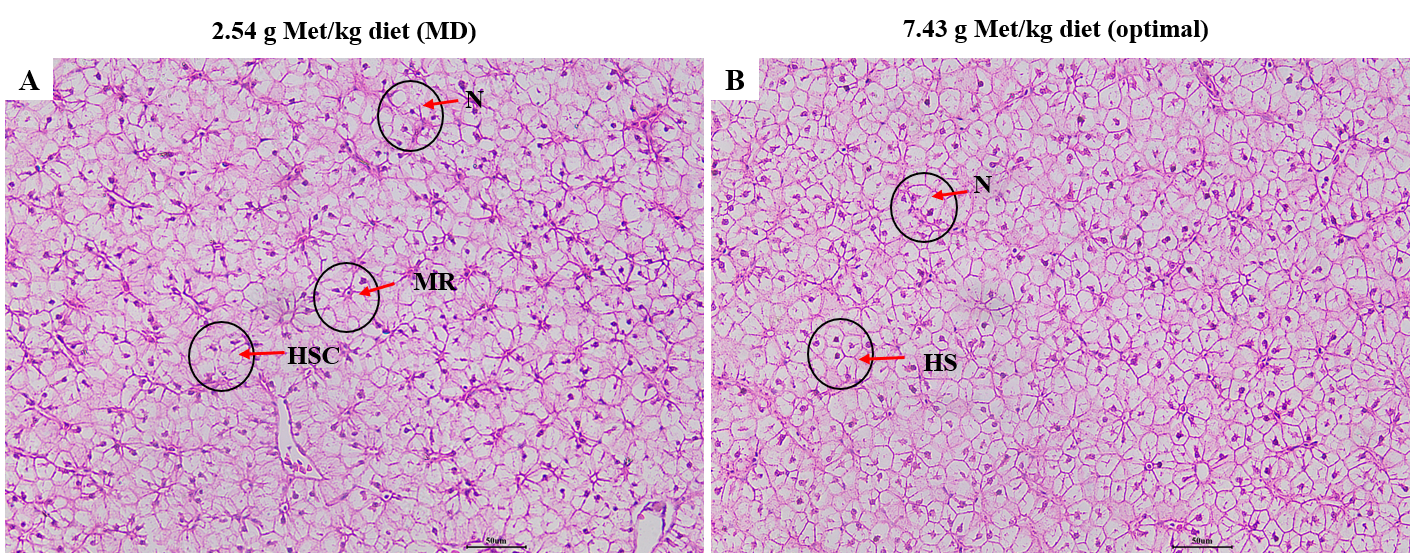


**Fig. S1** The histology of the of liver of grass carp after different concentrations of Met feeding for 60 d. Tissue sections of the liver of grass carp in MD group (**A**) and Met optimal group (**B**). The sections were stained with hematoxylin and eosin (H&E) in on-growing grass carp (*Ctenopharyngodon idella*) fed diets with specified levels of Met (g/kg) for 60 d (*n* = 3). Magnification ×200. MD, methionine deficiency; N, Nucleus, MR, Membrane rupture, HSC, Hepatic sinusoid congestion
